# Structural Simplification from Tricyclic to Bicyclic Scaffolds: A Long-Term Investigation in the Field of Adenosine Receptor Antagonists

**DOI:** 10.3390/cells14181480

**Published:** 2025-09-22

**Authors:** Costanza Ceni, Sara Calenda, Giulia Vagnoni, Daniela Catarzi, Flavia Varano, Vittoria Colotta

**Affiliations:** Dipartimento di Neuroscienze, Psicologia, Area del Farmaco e Salute del Bambino, Sezione di Farmaceutica e Nutraceutica, Università degli Studi di Firenze, Via Ugo Schiff, 6, 50019 Sesto Fiorentino, Italy; costanza.ceni@unifi.it (C.C.); sara.calenda@unifi.it (S.C.); giulia.vagnoni@unifi.it (G.V.); daniela.catarzi@unifi.it (D.C.); flavia.varano@unifi.it (F.V.)

**Keywords:** adenosine receptors, adenosine receptor antagonists, neuroinflammation, neuroprotection, antinociceptive, depression, cancer

## Abstract

Adenosine receptor (AR) antagonists have attracted considerable interest due to their therapeutic potential in a wide range of pathological conditions, including neurological, cardiovascular, and inflammatory disorders. Although a large number of AR antagonists have been developed worldwide, the interest in new derivatives remains high, and achieving subtype selectivity continue to be a major challenge. This review summarizes our research on adenosine receptor antagonists, highlighting the discovery of potent and selective compounds for the diverse AR subtypes across various chemical classes. Specifically, the paper focuses on the study of the triazolo[4,3-a]quinoxalin-1-one (**TQX**) and pyrazolo[3,4-c]quinoline (**PQ**) series, along with their simplified analogues, which have yielded highly potent and selective AR antagonists. An overview of the structure–activity relationship (SAR) studies and molecular docking investigations is provided, emphasizing the structural requirements for A_2A_ and A_3_ receptor–ligand interaction. In addition, we present pharmacological studies of selected AR antagonists, in various in vitro and in vivo models of pain, depression, neuroinflammation-related diseases, and cancer.

## 1. Introduction

Adenosine is a ubiquitous endogenous signaling molecule that modulates a wide variety of physiological functions [1].

**Figure 1:**
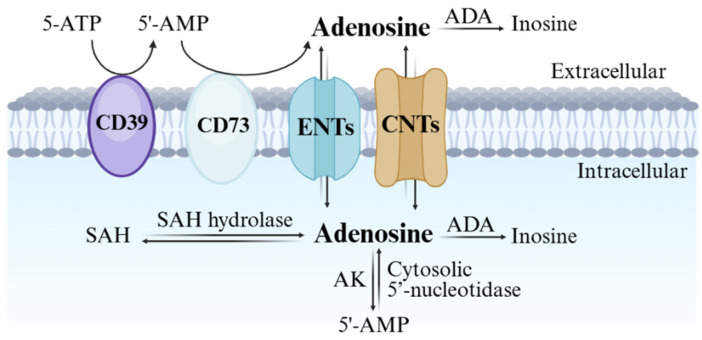
Biosynthesis and metabolism of adenosine.

Adenosine biosynthesis, metabolism, and transport are summarized in Figure 1. The nucleoside can be synthesized both in the intracellular and extracellular space. The major extracellular biosynthetic mechanism involves the degradation of ATP, ADP, and AMP, due to the activation of the ecto-nucleoside triphosphate diphosphohydrolase 1 (CD39) and the ecto-5’-nucleotidase (CD73) [2]. Nevertheless, under physiological conditions adenosine is primarily generated intracellularly through the hydrolysis of S-adenosylhomocysteine (SAH) by SAH hydrolase [3], and of AMP by endo-5’-nucleotidase [4].

Adenosine uptake or release from cells is regulated by the different concentrations on the two sides of the cell membrane and involves nucleoside transporters, which can be classified into equilibrative (ENT) and concentrative (CNT) nucleoside transporters [5]. While the four subtypes of ENTs (1–4) act through passive transport and facilitated diffusion, the Na^+^-dependent CNTs promote adenosine transport against its concentration gradient, by using the sodium ion gradient as an energy source. Normally, adenosine transport occurs from the extracellular to the intracellular milieu; however, the flow can be reversed under pathological conditions such as inflammation, hypoxia, and ischemia [6]. After intracellular uptake, adenosine is converted into inosine by adenosine deaminase (ADA) or phosphorylated to 5’-AMP through adenosine kinase (AK), providing a physiological half-life of less than 1s. The Michaelis constant (K_m_) values of these enzymes are 17–45 μM (ADA) and 2 μM (AK), suggesting AK is the major enzyme responsible for adenosine clearance, while deamination takes place preferably under pathological conditions [1].

### 1.1. Adenosine Receptors

**Figure 2:**
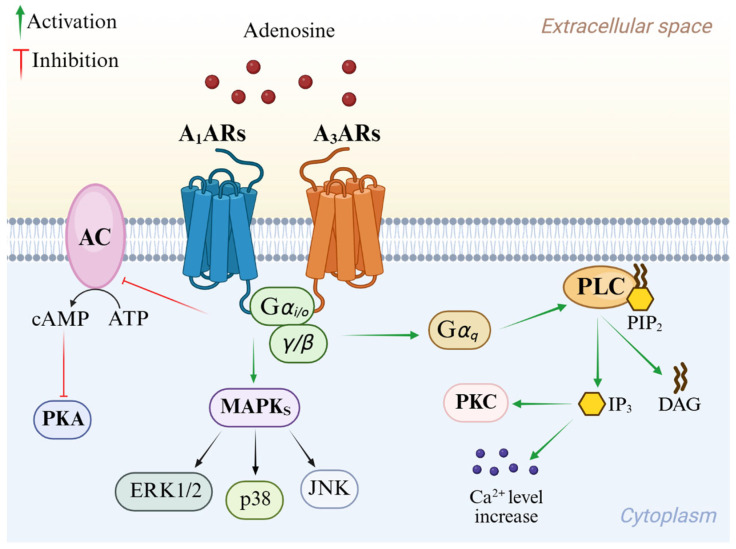
Overview of A_1_ AR and A_3_ AR intracellular signaling pathways.

Adenosine mediates its physiological effects by interacting with G protein-coupled receptors (GPCRs), classified into four subtypes: A_1_, A_2A_ AR, A_2B_ AR, and A_3_ AR adenosine receptors (ARs). All four ARs have been cloned and pharmacologically characterized [1]. All ARs modulate adenylyl cyclase (AC), thereby affecting cyclic AMP (cAMP) levels, but other effector systems have been observed [7]. Signal transduction pathways of A_1_ and A_3_ ARs are summarized in Figure 2. These AR subtypes are activated by nanomolar concentrations of adenosine [1] and preferentially couple to G_i_ and G_0_ proteins, thus decreasing AC activity and intracellular cAMP production. Additionally, they induce phospholipase C (PLC)-β activation, leading to an increase in inositol 1,4,5-trisphosphate (IP_3_), diacylglycerol (DAG), and intracellular Ca^2+^ levels, and activation of phosphokinase C (PKC) [1]. Activation of A_1_ and A_3_ ARs induces the intracellular phosphorylative cascade of the mitogen-activated protein kinase (MAPK) family, including MAPK p38, extracellular signal-regulated kinase 1/2 (ERK1/2), and Jun NH_2_-terminal kinase (JNK) [8,9,10]. A_3_ AR activation is also correlated to a different pathway from GPCR signaling which involves Ras homolog family member A (RhoA) and phospholipase D [11].

**Figure 3:**
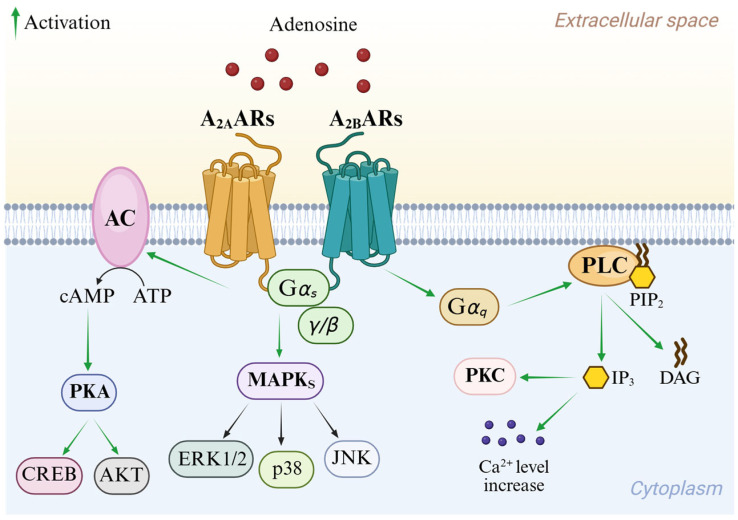
Overview of A_2A_ AR and A_2B_ AR intracellular signaling pathways.

Signal transduction pathways of the A_2A_ AR and A_2B_ AR are reported in Figure 3. Although they share almost identical binding sites, A_2A_ AR is activated by nanomolar concentrations of adenosine, while A_2B_ AR requires micromolar levels [12]. Both subtypes couple to G_s_ proteins, thereby activating AC and consequently leading to the enrolment of different cAMP-dependent effectors including protein kinase A (PKA). Through the activation of PKA and cross-talk with protein kinase B (AKT), these pathways modulate cell growth and proliferation via the element-binding protein (CREB) [1]. Moreover, A_2A_ and A_2B_ ARs are involved in the activation of MAPK signaling [4,13,14]. In addition, A_2B_ AR enrolment stimulates PLC through the G_q_ protein and regulates ion channels through its βγ subunits [4,14].

ARs are widely distributed throughout the body and their activation modulates several processes in different tissues and organs [15].

#### 1.1.1. A_1_ Adenosine Receptor

A_1_ AR is highly expressed in the central nervous system (CNS), taking part in the control of physiological synaptic transmission. Particularly, its activation reduces excitatory transmission through the enhancement of potassium current [16] and the inhibition of N-type calcium channels [17], thus leading to beneficial effects in several central disorders, including epilepsy and cerebral ischemia [18,19]. Additionally, several studies reported neuroprotective effects following A_1_ AR activation in various models of inflammatory and neuropathic pain, due to the involvement of the nitric oxide/cGMP/protein kinase G/KATP channel [20], as well as modulation of Ca^2+^ and K^+^ channels and inhibition of excitatory amino acid release [21]. A_1_ AR has been detected in β-amyloid plaques and neurons with tau deposition and its modulation ameliorates β-amyloid and tau neurotoxicity, thus improving cognition and memory. Thus, this receptor has been recognized to play a central role in the pathogenesis of Alzheimer’s Disease (AD) and its dysfunction may be responsible for some of the AD clinical manifestations [22]. A_1_ AR is also expressed in smooth muscle cells and cardiomyocytes in atria and ventricular tissues, taking part in the regulation of heart rate and blood pressure [23]. A_1_ AR activation appears to play a crucial role in myocardial ischemic preconditioning, a protective mechanism involving brief episodes of sub-lethal ischemia alternated with short periods of reperfusion prior to a prolonged ischemic insult [24]. In the kidney, it modulates glomerular filtration rate [25], while in adipose tissue it suppresses lipolysis [26]. Additionally, A_1_ AR activation inhibits glucose-stimulated insulin secretion from pancreatic β-cells [27].

#### 1.1.2. A_2A_ Adenosine Receptor

A_2A_ AR is the main AR subtype expressed in the striatum, where it co-localizes with dopamine D_2_ receptor (D_2_ R) to form A_2A_ AR/D_2_ R heteromers. These complexes play a critical role in motor function regulation. Notably, stimulation of A_2A_ AR/D_2_ R heteromers counteracts the D_2_ R-mediated inhibition of N-methyl-D-aspartate (NMDA) receptor activity [28], a mechanism implicated in the motor dysfunction characteristic of Parkinson’s disease (PD) [29]. Additionally, A_2A_ R has been also found in both pre- and post-synaptic neurons and in glial cells where it stimulates pro-inflammatory functions, particularly by inducing activation of both microglia and astrocytes in pro-inflammatory phenotypes [30,31]. Under physiological conditions, the A_2A_ AR expression in microglia and astrocytes is usually low, while it increases after brain insults, nerve injury, and inflammatory signals, taking part in an important feed-forward mechanism to locally control neuroinflammatory responses in the brain [32,33,34]. Therefore, modulation of A_2A_ AR could represent an innovative therapeutic strategy to counteract neurodegenerative disorders with neuroinflammatory background. A_2A_ AR is also involved in the development of neuropathic pain and its blockade confers protection [35]. Indeed, the changes in spinal cord excitability, which have been proposed to drive neuropathic pain, are closely linked to alterations in microglial functions [36]. A_2A_ AR is also located presynaptically on cortico-striatal glutamatergic afferents [37], where it modulates glutamate release [38,39]. Considering that several studies support the hypothesis that cortico-striatal glutamatergic deregulation could be involved in the pathogenesis of Huntington’s disease (HD) [40], A_2A_ AR has emerged as potentially druggable target in HD [41].

Peripherally, A_2A_ AR controls coronary vascular due to its expression in the smooth muscle and endothelium, where it induces vasodilation, thus providing a cardioprotective action [42]. Cardioprotective properties of A_2A_ AR also appear to be related to a reduction in neutrophil accumulation, due to its potent anti-inflammatory activity [43]. Furthermore, A_2A_ AR is largely expressed in immune system cells and its activation exerts opposite effects to the central ones, inhibiting T and natural killer lymphocytes, dendritic cells, monocytes, and macrophages [31].

#### 1.1.3. A_2B_ Adenosine Receptor

In the CNS, A_2B_ AR is mostly located in astrocytes, where its expression is upregulated in response to lipopolysaccharide (LPS) and hypoxic stimulation [44]. At this level, A_2B_ AR stimulation induces astrogliosis [45], which is strongly linked to neurodegenerative processes [46]. Moreover, A_2B_ AR contributes to the modulation of the inflammatory cascade and neuronal injury following global cerebral ischemia [47].

At the peripheral level, A_2B_ AR is widely expressed in the bladder [48], intestine [49], and fibroblasts [50]. Several lines of evidence have highlighted a pronociceptive and proinflammatory role for this receptor, making it a promising therapeutic target for a range of inflammatory diseases, including multiple sclerosis (MS) [47], wound healing disorders, fibrosis, asthma, colitis, and diabetes [51]. Furthermore, A_2B_ AR activation enhances the growth of multiple cancer cell lines and contributes to solid tumor development, by inhibiting anti-tumor immune responses. Due to its low affinity for adenosine, the receptor is primarily activated in pathological conditions where extracellular adenosine levels are elevated, such as within tumors [52].

#### 1.1.4. A_3_ Adenosine Receptor

A_3_ AR is widely distributed in the human body. Low levels have been reported in several brain areas, including the cortex, hippocampus, thalamus, hypothalamus, retinal ganglion cells, and motor nerve terminals [53]. A_3_ AR is expressed in astrocytes and microglia [54], where it affects, respectively, the expression of proinflammatory genes, including those for inducible nitric oxide synthase [44], and regulates TNF-α production [55,56,57]. This suggests an anti-inflammatory role of this AR subtype in the CNS. Moreover, several studies highlighted A_3_ AR as an antinociceptive drug target [58], especially in neuropathic pain. At this level, A_3_ AR stimulation provided inhibition of the mechanoallodynia induced by the chronic constriction injury of the sciatic nerve, increasing at the same time the potency of classical analgesic drugs [59,60].

At the peripheral level, A_3_ AR is located in epithelial cells, respiratory apparatus, and cardiovascular system [1]. In the latter, A_3_ AR is greatly expressed in the coronary and carotid arteries [61]. However, it is well known that A_3_ AR activation provides cardioprotective effects through the reduction of inflammatory responses and the resultant apoptotic signals [62,63]. Furthermore, A_3_ AR has been found in inflammatory cells, such as macrophages, neutrophils, eosinophils, lymphocytes, mast cells, and osteoblasts, where it regulates anti-inflammatory processes [7]. Interestingly, A_3_ AR is overexpressed in several cancer cells and tissues, and both its blockade or activation produce an antitumor effect, suggesting that it might be a theranostic target in different type of cancer [64,65,66].

#### 1.1.5. Adenosine Receptor Heteromers

AR subtypes can form supramolecular complexes—heteromers—through interactions with other GPCRs, often acquiring distinct functional properties. These complexes are relevant when considering ligand binding and downstream signaling [67,68].

The A_2A_ AR is highly expressed in the striatum, where it co-localizes with the dopamine D_2_ R to form A_2A_ AR/D_2_ R heteromers [28,29]. A_2A_ AR also interacts with the cannabinoid type 1 receptor (CB_1_ R), forming A_2A_ AR/CB_1_ R heteromers in the striatum and hippocampus. In the striatum, this complex modulates cannabinoid-induced psychomotor effects, with A_2A_ AR overexpression leading to a downregulation of cannabinoid signaling [69]. However, under certain conditions, presynaptic A_2A_ AR blockade may facilitate CB_1_ R signaling underscoring the need for context-specific evaluation [70]. In microglia, A_2A_ AR forms heteromers with cannabinoid type 2 receptor (CB_2_ R), where A_2A_ AR antagonists enhance CB_2_ R signaling and exert anti-inflammatory effects [71,72]. Another relevant complex is the heteromer of A_1_ AR with dopamine D_1_ receptor (D_1_ R) which is expressed in spinal motoneurons and reduces excitability through inhibition of adenylyl cyclase [73]. The A_1_/A_2A_ AR heteromer in astrocytes acts as a molecular switch, favoring activation of one receptor subtype over the other depending on extracellular adenosine levels [68]. The formation of A_2A_/A_2B_ receptor heteromers has been reported in various tissues, where it markedly diminishes A_2A_ receptor-mediated signaling and decreases its affinity for agonists [68]. These findings have important implications for drug research, as compounds with high in vitro affinity for the A_2A_ AR may became ineffective in the presence of A_2A_/A_2B_ AR heteromers. A_2A_ AR antagonists, in particular, may exhibit altered behavior when targeting these heteromeric complexes. In some cases, their use has shown therapeutic benefits by modulating partner receptor activity, such as enhancing D_2_ R-mediated effects [28,29].

### 1.2. Therapeutic Potential of AR Agonists

As previously discussed, AR signaling plays a pivotal role in numerous physiological and pathological processes. Consequently, its therapeutic modulation has emerged as a promising strategy to counteract a variety of diseases, especially those characterized by inflammation, immune dysfunction, and tissue injury.

Selective AR agonists, particularly those targeting the A_2A_ and A_3_ ARs, are currently under active clinical investigation for conditions marked by excessive inflammation, immune dysregulation, or tissue injury [74]. Among them, A_2A_ AR agonists have attracted significant attention due to their potent anti-inflammatory and vasodilatory effects. The A_2A_ agonist **regadenoson**, already approved for pharmacologic cardiac stress testing, is being repurposed in early-phase clinical trials for COVID-19, myocardial ischemia, lung transplantation, and high-grade gliomas [75,76,77,78,79,80]. These conditions share common pathophysiological features, including hypoxia-driven inflammation and immune activation, in which A_2A_ AR stimulation may help limit tissue damage and promote homeostatic resolution.

In parallel, A_3_ AR agonists have demonstrated therapeutic potential in various immune-mediated and proliferative disorders, such as plaque psoriasis, hepatocellular carcinoma (HCC), and non-alcoholic steatohepatitis (NASH). For instance, **piclidenoson** (**CF101**), currently in phase III clinical trials for psoriasis, has shown anti-inflammatory activity by downregulating pro-inflammatory cytokines, including TNF-α and IL-17 [81]. Another A_3_ AR agonist, **namodenoson** (**CF102**), has reached phase III trials for HCC in patients with cirrhosis and is also being investigated for NASH [82,83]. Additionally, the A_3_ AR agonist **FM-101** is under evaluation in early-phase studies for ocular hypertension and NASH [84], further illustrating the pharmacological versatility of this receptor subtype as a therapeutic target.

### 1.3. Therapeutic Potential of AR Antagonists

Multiple lines of evidence have shown that blockade of AR signaling by AR antagonists can have a favorable impact on the onset and progression of various pathological conditions. In fact, AR antagonists have demonstrated significant therapeutic potential, driving the development of numerous such compounds worldwide offering promising avenues for the treatment of a range of diseases including respiratory and neurodegenerative disorders, neuropathic pain, and cancer [85,86].

The A_1_ AR antagonist **PBF-680** is under clinical investigation for asthma and chronic obstructive pulmonary disease (COPD), based on its capacity to reduce adenosine-induced bronchoconstriction and inflammation [87].

**Figure 4:**
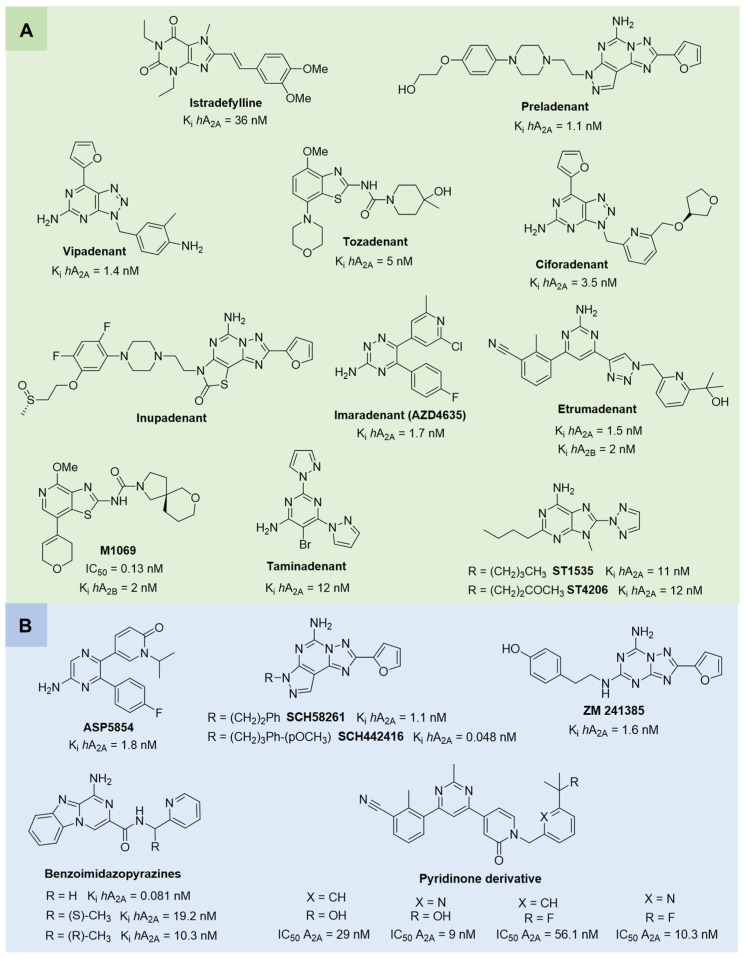
(**A**) Structures of A_2A_ AR antagonists and dual A_2A_/A_2B_ AR antagonists undergoing clinical trials. (**B**) Structures of A_2A_ AR antagonists used as pharmacological tools or showing promising activity in preclinical models of neurodegenerative diseases and cancer.

A_2A_ AR antagonists have been primarily investigated in neurodegenerative disorders and oncology [72]. The selective A_2A_ AR antagonist **istradefylline** (Figure 4), already approved in Japan (since 2013) and the United States (since 2019) for the treatment of PD [88], is currently being investigated for additional neurological indications, such as cognitive impairment and apathy in PD patients, as well as neuroprotective effect in an animal model of experimental autoimmune encephalomyelitis (EAE) [89,90]. Among A_2A_ AR antagonists, **preladenant** (Figure 4) emerged as one of the most promising compounds for PD. It demonstrated favorable outcomes in both preclinical and early-phase clinical studies, improving motor symptoms without exacerbating dyskinesias when administered in combination with L-DOPA. However, despite this initial promise, phase III trials failed to meet their primary efficacy endpoints, ultimately resulting in the withdrawal of the compound from further development [91,92,93,94]. **Vipadenant** (Figure 4) was one of the earliest selective A_2A_ AR antagonists investigated in clinical trials for PD. Nerveless, its development was halted after phase II due to insufficient efficacy and safety concerns [95]. Similarly, **tozadenant** demonstrated effectiveness in reducing “off” time in PD patients when used as an adjunct to L-DOPA. However, clinical development was discontinued following reports of severe adverse events, including agranulocytosis [96,97,98].

Another noteworthy class of A_2A_ AR antagonists includes compound **ST1535** and its metabolite **ST4206** (Figure 4), which exhibited anti-Parkinsonian activity in rodent models. Notably, **ST1535** counteracted dopaminergic neuron degeneration and glial activation, while concurrently improving motor deficits when administered alongside low doses of L-DOPA. These promising preclinical findings prompted the advance of **ST1535** and its metabolites into a phase I clinical trial, where the compound demonstrated a favorable safety and tolerability profile [99,100,101,102]. In addition, the dual A_1_/A_2A_ receptor antagonist **ASP5854** (Figure 4) has been evaluated in preclinical models of PD, showing neuroprotective and motor symptom-improving effects [103,104].

In parallel, novel A_2A_ AR antagonists, including **ciforadenant, inupadenant**, **imaradenant** (**AZD4635**), and **taminadenat** (Figure 4), have been evaluated in early-phase trials for various solid tumors. These agents aim to restore anti-tumor immunity by blocking adenosine-mediated immunosuppression within the tumor microenvironment [86,105,106,107,108,109,110,111]. Aligned with this immunotherapeutic strategy, dual A_2A_/A_2B_ AR antagonists, **etrumadenant** and **M1069** (Figure 4), have being investigated across multiple cancer types, including prostate, colorectal, urothelial, and head and neck tumors, progressing to phase II trials. The simultaneous blockade of A_2A_ and A_2B_ receptors is intended to enhance immune cell responsiveness and function, thereby counteracting the immunosuppressive effects driven by hypoxic and adenosine-rich tumor environments [112,113]. Several compounds, including **SCH58261** and **SCH442416** (Figure 4), have been widely used in experimental studies to elucidate A_2A_ AR pharmacology and downstream signaling pathways. They have been instrumental in clarifying the functional role of A_2A_ AR across various pathological conditions. In particular, **SCH58261** was shown to improve cognitive function in Alzheimer’s disease model mice by activating Nrf2 via autophagy and to attenuate neuroinflammation in a mouse model of EAE by reducing microglia/macrophage activation and T cell infiltration [114,115,116,117,118]. Additional antagonists, including the potent and selective A_2A_ AR antagonist **ZM241385** (Figure 4), have primarily been employed in research settings to investigate the roles of adenosine signaling in neurodegeneration and cancer immunology [119,120,121,122]. Recently, two novel classes of A_2A_ AR antagonists have been identified for cancer immunotherapy. The first one includes benzo[4,5]imidazo[1,2-a]pyrazin-1-amine derivatives (Figure 4), which display subnanomolar affinity for the A_2A_ AR and exhibit potent immunostimulatory activity. Compared to the clinical candidate **AZD4635**, these compounds more effectively reversed the immunosuppressive tumor microenvironment, thereby inhibiting tumor growth in preclinical models, without signs of toxicity at the tested doses [123,124]. The second class includes pyridinone-based A_2A_ AR antagonists, identified through high-throughput screening and structure–activity relationship (SAR) optimization. These compounds demonstrated potent receptor antagonism, favorable pharmacokinetic properties, and the ability to enhance T cell activation by modulating key immunoregulatory pathways. In preclinical tumor models, they showed significant antitumor activity, supporting their development as potential candidates for cancer immunotherapy [125].

Due to the critical role of A_2B_ AR in regulating immune responses, inflammation, and tissue remodeling within the tumor microenvironment, recently A_2B_ AR selective antagonists have emerged as promising therapeutic agents in oncology. These antagonists are particularly valued for their ability to modulate the tumor microenvironment by suppressing immunosuppressive signaling pathways, inhibiting tumor growth, and reducing angiogenesis. However, to date, the number of highly selective A_2B_ AR antagonists remains limited. Furthermore, the evaluation of A_2B_ AR signaling in specific tumor types is challenging due to the receptor’s low affinity for endogenous adenosine under physiological conditions. Additionally, its expression is highly context-dependent, varying significantly with physiological or pathological states such as hypoxia and inflammation, which further complicates preclinical characterization and therapeutic targeting [126,127].

**Figure 5:**
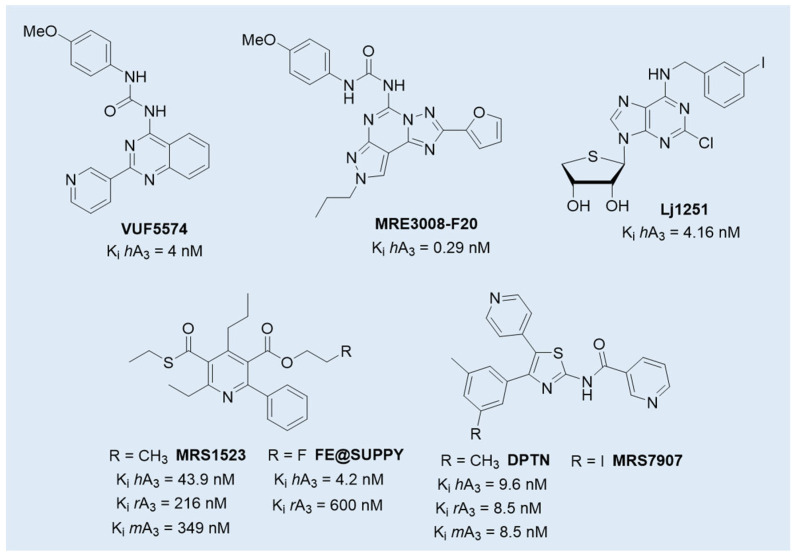
Structures of selected A_3_ AR antagonists with preclinical efficacy or cross-species activity.

Although still in early phases, A_3_ AR antagonists **PBF-1650** and **PBF-677**, whose structures have not, to the best of our knowledge, been publicly disclosed [128], are under investigation for immune-mediated inflammatory conditions, including psoriasis and ulcerative colitis, expanding the therapeutic potential of this class [74]. Their clinical relevance is further supported by preclinical studies in additional pathological contexts such as neurodegenerative diseases, traumatic brain injury, and cancer, where A_3_ AR signaling has been implicated [11,58,108,110]. Among the selective A_3_ AR antagonists, compounds such as **VUF5574**, **MRE3008-F20**, and **LJ1251** (Figure 5) have been extensively characterized for their pharmacological profiles and potential clinical applications. **VUF5574** and **MRE3008-F20** exhibited high affinity and selectivity for the human A_3_ AR, effectively modulating receptor activity in preclinical models, making it a valuable tool in dissecting receptor function and signaling dynamics [129,130,131,132]. Moreover, the human A_3_ AR antagonist **LJ1251** demonstrated a remarkable neuroprotective effect in a cellular model of ischemia, preventing ischemic neuronal injury induced by severe OGD [133].

However, despite this promising therapeutic potential, significant challenges remain in translating preclinical findings for A_3_ AR antagonists from animal models to humans. One major obstacle lies in the notable interspecies differences in receptor sequence and structure. The amino acid sequence identity between rat and human A_3_ AR is approximately 72–75%, a relatively low value compared to other adenosine receptor subtypes [134]. Crucially, these differences affect specific receptor domains involved in antagonist binding, while the regions critical for agonist interaction are more conserved across species. As a result, most A_3_ AR antagonists at the human receptor exhibit reduced or no activity in rodent models, leading to potentially misleading preclinical data [134,135,136]. Only few compounds preserve activity across species, including **DPTN** (and its 3-iodo analogue **MRS7907**), **MRS1523,** and **FE@SUPPY** (Figure 5). DPTN displayed nanomolar affinity for human (K_i_ = 1.6 nM), mouse (K_i_ = 9.6 nM), and rat (K_i_ = 8.5 nM) A_3_ ARs, but moderate selectivity against other AR subtypes [136,137]. **MRS1523** maintained cross-species antagonism but with significantly lower affinity at rodent receptor (K_i_ = 43.9 nM in human, 349 nM in mouse, and 216 nM in rat) and limited subtype selectivity, especially at higher concentrations [138,139]. The PET tracer **FE@SUPPY** (unlabeled form), structurally related to MRS1523 [138], was used to label rat brain A_3_ ARs. The autoradiography was performed on rat brain slices in the presence or absence of Cl-IB-MECA, suggesting that it has the potential to serve as the first positron emission tomography (PET) tracer for the A_3_ AR, even if the outcomes did not yield definitive conclusions [140].

The ongoing trials underscore the versatility of AR antagonists as potential drugs, indicating that development of novel more selective, potent, and pharmacokinetically optimized compounds remains a critical challenge.

## 2. Structural Simplification from Tricyclic to Bicyclic Scaffolds in the Design of Adenosine Receptor Antagonists

This review presents a comprehensive overview of about twenty-five years of our research in the field of adenosine receptor antagonists, which has led to the discovery of numerous potent compounds across various chemical classes, characterized by high selectivity for the different AR subtypes. The new derivatives were designed following general principles of medicinal chemistry, with the most recent work focusing on molecular simplification to obtain compounds with improved physicochemical properties.

**Figure 6:**
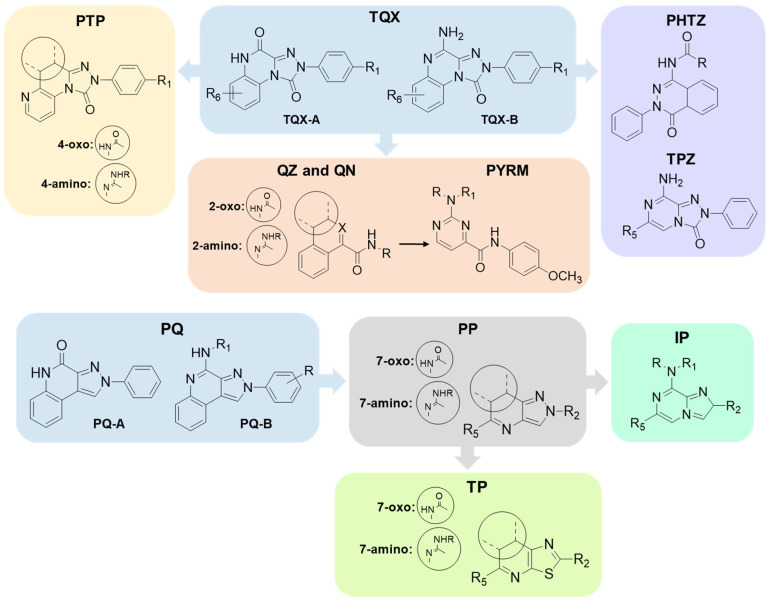
The herein reported tricyclic AR antagonists and their simplified analogues.

Specifically, this review focuses on different heterocyclic classes of AR antagonists that were designed starting from two tricyclic series, the 1,2,4-triazolo[4,3-a]quinoxalin-1-ones (**TQX**) and the pyrazolo[3,4-c]quinolines (**PQ**). Both the series were deeply investigated and from each of them simplified analogues were designed leading to bicyclic and monocyclic ligands (Figure 6). For each class of ligands, structure–activity relationships (SARs) are discussed, highlighting the structural features required for affinity and selectivity toward the different AR subtypes. The results of molecular docking investigation are reported to describe the hypothetical binding pose of the ligands at the AR recognition sites. Additionally, the results of in vitro and in vivo pharmacological studies are presented, emphasizing the neuroprotective, antinociceptive, antiproliferative, and antidepressive effects of AR antagonists.

### 2.1. 2-Aryl-1,2,4-triazolo[4,3-a]quinoxalin-1-one Derivatives and Their Simplified Analogues

#### 2.1.1. 2-Aryl-1,2,4-triazolo[4,3-a]quinoxalin-1-one Derivatives

**Figure 7:**
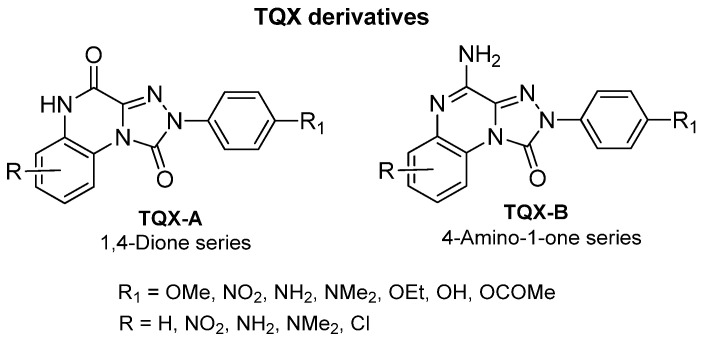
2-Aryl-1,2,4-triazolo[4,3-a]quinoxalin-1-one (**TQX**) derivatives.

This section presents the study of 2-aryl-1,2,4-triazolo[4,3-a]quinoxalin-1-one derivatives, classified into the 1,4-dione and the 4-amino-1-one series (**TQX**-**A** and **TQX-B**, respectively, Figure 7). Notably, the triazoloquinoxalin-1,4-dione scaffold (**TQX-A**) emerged as a valuable framework for the development of potent and selective A_3_ AR antagonists. In contrast, the 4-amino-triazoloquinoxalin-1-one core (**TQX-B**) gave rise to potent and selective antagonists of A_1_, A_2A_, or A_3_ AR subtypes, depending on the substitution pattern on the aromatic rings and the 4-amino group [141,142]. Finding new potent antagonists for A_3_ AR within the **TQX**-**A** series was of high interest, as the involvement of this AR subtype in regulating several pathological processes, including neuronal inflammation, immune responses, and cardiac function, was becoming increasingly evident. Therefore, selective A_3_ AR antagonists were sought both as pharmacological probes to elucidate receptor-mediated pathophysiological mechanisms and as candidates for therapeutic intervention in autoimmune diseases, cancer, brain injury, and neurodegenerative disorders [13,56,74,143,144].

##### 2-Aryl-1,2,4-triazolo[4,3-a]quinoxalin-1,4-dione Derivatives

The study of the **TQX-A** series (Figure 7) was encouraged by the interesting profile of the unsubstituted compound **1** (Table 1), which showed nanomolar A_3_ AR affinity (K_i_ = 80 nM) and high selectivity versus the A_1_ and A_2A_ ARs. Thus, various substituents were inserted on the 2-phenyl ring (R_1_) and/or on the fused benzo moiety (R_6_, R_8_) to improve A_3_ affinity and selectivity of derivative **1** [141,145,146].

Among the R_1_ substituents, characterized by varying electronic properties, lipophilicity, and steric bulk (OMe, OH, OEt, OCOMe, NO_2_), the most effective were the methoxy (compound **2**, K_i_ = 16 nM) [141] and the nitro (compound **3**, K_i_ = 0.6 nM) [146]. Compound **3** displayed antagonist activity in [^35^S]GTPγS binding assays at the hA_3_ AR (EC_50_ = 5.4 nM), making it one of the most potent hA_3_ AR antagonists reported to date, with high selectivity versus hA_1_ and hA_2A_ AR subtypes. Notably, since the most active derivatives bear either electron-donating (e.g., OMe) or electron-withdrawing (e.g., NO_2_) substituents, it was inferred that the electronic nature of the R_1_ group does not critically influence receptor binding.

Docking studies at a homology model of the hA_3_ AR depicted the putative binding poses of these derivatives [146], which presented the fused benzo ring located in a tiny hydrophobic pocket delimited by transmembrane (TM) domains 5 and 6, and the 2-aryl ring positioned in a small hydrophobic cavity delimited by extracellular loop 2 (EL2), and by non-polar amino acids residues, such as Leu90 (TM3), Leu246 (TM6), and Ile268 (TM7). At least two H-bonding interactions were evidenced, the first between the carbonyl group at the 1-position and the NH of the Gln167–Phe168 amide bond. The 1-carbonyl group could also interact with the amide moiety of Asn250 side chain, a residue conserved among all AR subtypes, and important for ligand binding. Steric factors and the ability of the R_1_ group to act as a hydrogen bond acceptor were relevant for receptor interaction [146]. In particular, the para-nitro group of derivative **3** strongly achieved two hydrogen-bonding interactions with Ser165 (EL2) and His272 (TM7) which could be responsible for the observed subnanomolar activity at the hA_3_ AR. Lipophilic chlorine atom(s) and polar or hydrophilic groups (NH_2_, NO_2_), at the 6,8-positions of the fused benzo ring, were not well tolerated by the A_3_ AR, and also negatively affected affinity for the A_2A_ AR, while increasing the A_1_ AR binding [145]. When R_1_ = OMe was combined with R_6_ = NO_2_ (derivative **4**), higher A_3_ AR affinity was found with respect to that of the p-methoxy derivative **2**.

##### 4-Amino-2-aryl-1,2,4-triazolo[4,3-a]quinoxalin-1-one Derivatives

The structural modification performed on **TQX-A** were applied also to the 2-aryl-1,2,4-triazolo[4,3-a]quinoxalin-4-amino-1-one series (**TQX-B**, Figure 7) [141,145,146]. Overall, these 4-amino derivatives demonstrated the ability to bind A_1_, A_2A_, and A_3_ ARs with affinities ranging from subnanomolar to high nanomolar values (Table 2). The unsubstituted derivative **5** showed high affinity for A_1_ and A_2A_ ARs, and more than 10-fold lower affinity for the A_3_ AR subtype. Compounds bearing a substituent on the 2-phenyl group (R_1_) or on the fused benzo ring (R_6_, R_7_, and R_8_) generally displayed reduced affinity for A_1_ and A_2A_ ARs, with respect to compound **5**, with the exception of derivatives **12**, **13**, which showed, respectively, similar and better affinity for the bA_1_ AR, compared to **5**, compound **13** being the most active (K_i_ = 0.2 nM). The ability of the R_1_ substituent to engage in a hydrogen bond, as well as its steric properties, play a role in hA_3_ receptor recognition. The most beneficial group for A_3_ AR affinity and selectivity was R_1_ = OMe (compound **6**) while R_1_ = NO_2_ was deleterious (compound **7**), in contrast to the effect elicited in the corresponding 1,4-dione derivative **3** (Table 1).

Lipophilic chlorine atom(s) and polar or hydrophilic groups (NH_2_, NO_2_) at the 6-, 7- and 8-positions (derivatives **8**–**13**, Table 2) [145] significantly enhanced hA_3_ AR affinity, compared to the lead **5**. In particular, the 6-NO_2_ group afforded the highest hA_3_ AR affinity (compound **10**, K_i_ = 4.75 nM). Combination of R_6_ = NO_2_ or NH_2_ with R_1_ = OMe (derivatives **9** and **10**) maintain a high A_3_ AR affinity (K_i_ = 47 nM, and 22 nM, respectively). Insertion of a chloro substituent at positions 7 or 8 was also favorable (derivatives **11** and **12**), The combination of an 8-Cl with a 6-NO_2_ significantly enhanced A_3_ AR affinity, yielding derivative **13**, the most active of this set (K_i_ = 0.112 nM).

##### 4-Amido-2-aryl-1,2,4-triazolo[4,3-a]quinoxalin-1-one Derivatives

The presence of acyl substituents on the 4-amino group yielded **TQX** derivatives exhibiting nanomolar affinity and high selectivity for the hA_3_ AR (compounds **14**–**23**, Table 3), in accordance with the results observed in other series of antagonists of similar size and shape [147,148]. Acyl substituents with varying steric bulk were investigated either alone or in combination with the beneficial substituents on the 2-phenyl ring (R_1_ = OMe), and the fused benzo ring (R_6_ = NO_2_, NH_2_) [149]. The presence of R_1_ = OMe significantly increased hA_3_ versus hA_1_ selectivity, while maintaining high hA_3_ AR affinity (compare **17**, **20**, and **23** to **15**, **16**, and **22**, respectively). The contemporary presence of R_1_ = OMe and R_6_ = NO_2_ dropped hA_3_ AR affinity to the micromolar range with the sole exception of the 2-diphenylacetamido derivative **21**, highly active (K_i_ = 0.8 nM) and selective. Interestingly, double substitution with R_1_ = OMe and R_6_ = NH_2_ yielded compounds with high hA_3_ AR affinities (K_i_ = 1–5.5 nM) and selectivity (e.g., **19**), regardless of the acyl group at R_4_.

Derivatives **19**, **20**, **22**, and **23**, tested in cAMP functional assays, proved to be potent antagonists at the hA_3_ AR (IC_50_ = 8.6–31.8 nM), and inactive at the hA_2B_ AR (IC_50_ > 1000 nM) [149].

Summarizing, the 4-methoxy group on the 2-phenyl ring, acyl moieties on the 4-amino group or a 6-nitro substituent on the benzo-fused ring provided derivatives endowed with nanomolar affinity and high selectivity for hA_3_ AR [141,145,146,149]. Molecular modeling studies at a homology model of the hA_3_ AR evidenced hydrogen-bonding interactions, involving the 1-carbonyl, the R_1_ = OMe, the R_6_ = NO_2_, and the 4-acylamino chain [149]. In particular, the 4-NH–CO moiety was surrounded by the polar amino acids Thr94 (TM3), His95 (TM3), and Ser247 (TM6). The hydroxyl residue of the latter was appropriately oriented to form an H-bond with the carbonyl oxygen of the 4-NHCO group, which in turn played a key role in orienting the R_4_ substituent within a hydrophobic pocket.

Given the critical role of the R_4_ group for hA_3_ AR affinity, other acyl substituents and sulfonyl (SO_2_Me, SO_2_Ph) or carbamoyl (CONHCOR) groups were introduced on the 4-amino residue. Additionally, 4-OCH_2_Ph-substituted derivatives were synthesized to assess the importance of the 4-NHCOR group for the anchoring at the hA_3_ AR [150]. The 4-pyridylcarbonyl-substituted compound **24** (Table 4) was one of the most active derivatives, exhibiting a nanomolar hA_3_ AR affinity and an enhanced selectivity, with respect to the benzoyl derivative **15** (Table 2). The 4-NHSO_2_Ph derivative **25** also showed high hA_3_ AR affinity and selectivity. Insertion of two SO_2_Me groups on the 4-amino moiety gave compound **27** which displayed nanomolar hA_3_ AR affinity (K_i_ = 5.5 nM), high hA_3_ AR antagonistic potency (IC_50_ = 12 nM, cAMP assay), as well as high selectivity versus the other hARs. Benzylureido group in R_4_ yielded good affinity for the hA_3_ AR (compound **28**) and even better for the hA_1_ AR. It is notable that the 4-benzyloxy derivative **29** possessed high affinity and selectivity for hA_3_ AR (K_i_ = 21 nM), although lacking the 4-NH–CO–group. The beneficial effect of the R_1_ = OMe was confirmed for derivatives **26** and **30** showing higher hA_3_ AR affinities compared to their unsubstituted counterparts **25** and **29**. These new derivatives served as molecular probes to construct refined models of the hA_3_ AR developed through a ligand-based homology modeling (LBHM) approach. Docking of derivatives **27** and **28** induced a different reorganization of the binding site resulting in molecular volumes of the TM binding cavity of 1120 Å^3^ and 980 Å^3^, respectively [150]. The network of interactions observed was consistent with that above described for derivatives **14**–**23**.

Derivative **27** was tested in an in vitro electrophysiological rat model of cerebral ischemia, obtained by oxygen and glucose deprivation (OGD). During an ischemic stroke, the levels of adenosine increase, activating the harmful A_3_ AR subtype both in vitro and in vivo [151,152]. Thus, targeting this AR subtype could be useful for the treatment of the pathology. Derivative **27**, at 10 nM concentration, delayed the onset of anoxic depolarization, a sign of brain damage, demonstrating its effectiveness in enhancing the resistance of the CA1 region of the hippocampus to ischemic injury. This result contrasted with the very low affinity of **27** at the rat A_3_ AR (% inhibition (I%) @ 1 µM = 5%) but was consistent with the results obtained with other hA_3_ AR antagonists belonging to diverse chemical series which showed high affinity for the hA_3_ AR and very low affinity for the rat A_3_ AR [133,153,154], due to the well-known low homology between the two receptors [135,136].

**Figure 8:**
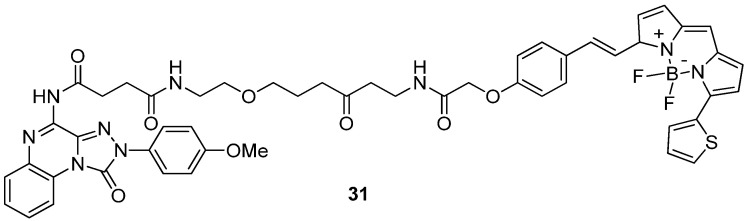
Structure of the fluorescent highly potent and selective hA_3_ AR antagonist **31**.

The robustness and versatility of the 4-amino-triazoloquinoxalinon-1-one scaffold to develop hA_3_ AR antagonists were recognized by Vernall et al. [155] who selected derivative **6** (Table 2) as the lead for the development of fluorescent antagonists to be used as molecular probes for the study of the A_3_ AR. Various fluorophores were introduced at the 8-amino position of **6** through linkers of different natures. The most promising compound was the BODIPY-X-630/650-conjugated derivative **31** (Figure 8) exhibiting high affinity for the hA_3_ AR (pK_D_ = 9.36 ± 0.16) and over 650-fold selectivity versus other ARs. The compound possessed excellent imaging properties, making it a valuable tool for investigating A_3_ AR.

##### 6-(Hetero)arylalkylamino-1,2,4-triazolo[4,3-a]quinoxalin-1-one Derivatives

**Figure 9:**
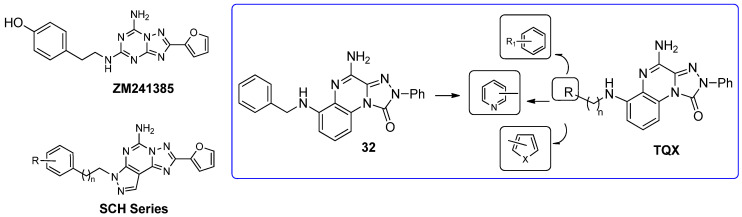
Structures of known A_2A_ AR antagonists and 6-(hetero)arylalkylamino-**TQX** derivatives.

Some investigations on the **TQX-B** series focused on the development of hA_2A_ AR antagonists, which were designed taking the well-known A_2A_ AR antagonists **ZM241385** [156] and derivatives belonging to **SCH** series as lead compounds (Figure 9) [157,158]. The 4-amino-6-benzylamino-2-phenyl-substituted **TQX** derivative **32** was the first to be synthesized (Figure 9), resulting in a potent and selective hA_2A_ AR antagonist [141,142]. Polar substituents capable of forming hydrogen bonds (F, OCH_3_, OH, COOH) were introduced on the 6-benzylamino moiety since they might enhance both hA_2A_ AR affinity and the physicochemical properties of the compounds, such as water solubility [142]. The carboxy group afforded the best hA_2A_ AR affinity (Table 5, derivative **33**). Even if some of the new compounds exhibited nanomolar hA_2A_ AR affinity, none of them exceeded the hA_2A_ AR affinity and selectivity of the parent compound **32**. The replacement of the phenyl moiety of **32** with less hindered bioisostere furyl or thienyl rings gave the best binding affinity results (compounds **34**, **35**).

#### 2.1.2. 2-Arylpyrido[2,3-e]-1,2,4-triazolo[4,3-a]pyrazin-1-one Derivatives

As discussed above, the presence of R_6_ = NO_2_ in the **TQX** series often favors anchoring to the hA_3_ AR through H-bond formation but in some 6-nitro-4-amido-substituted derivatives unfavorable steric interactions between the nitro group and the receptor site could occur, leading to a reduced affinity. To overcome this drawback, a series of 2-arylpyrido[2,3-e]-1,2,4-triazolo[4,3-a]pyrazin-1-one (**PTP**) was designed (Table 6), in which the endocyclic nitrogen atom intended to serve as an H-bond acceptor, in the place of the 6-nitro group, without causing steric hindrance [159].

The unsubstituted compound **36** exhibited quite low affinity for the hA_3_ AR (K_i_ = 656 nM), while it was active at the bA_1_ and bA_2A_ ARs. As in the **TQZ** lead, the presence of R_1_ = OMe enhanced hA_3_ AR affinity and selectivity (Table 6, derivative **37**). The same applies to the effect of acyl residues on the 4-amino group (R = COMe, COPh, COCH_2_Ph), especially in combination with R_1_ = OMe, as demonstrated by compound **39**, which showed the highest hA_3_ AR affinity (K_i_ = 4.54 nM) and selectivity versus both hA_1_ and hA_2A_ ARs. The compound was tested in cAMP assays, proving to be a potent antagonist at the hA_3_ AR (EC_50_ = 36.3 nM) and inactive at the hA_2B_ subtype (IC_50_ > 10,000 nM).

Compound **39** resulted to be effective in an in vitro rat model of cerebral ischemia, since it prevented the failure of synaptic activity induced by OGD in the hippocampus. Notably, this compound exhibited nanomolar affinities for the hA_3_ receptor, but showed no binding activity at the rA_3_ AR, in accordance with the findings above discussed for derivative **27** [150].

#### 2.1.3. From the Tricyclic **TQX** to the 2-Oxo/2-Aminoquinazoline-4-carboxamide Scaffold

**Figure 10:**
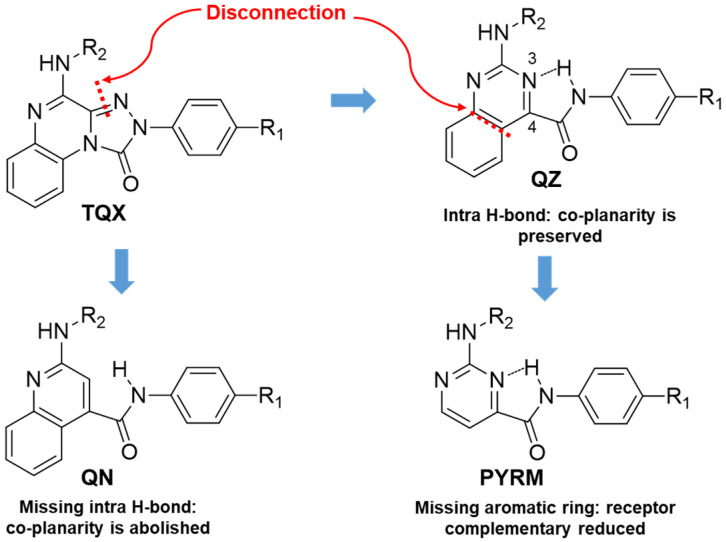
Design of the **TQX** analogues through molecular simplification approach.

An in silico molecular simplification approach was employed to design three series of **TQX** analogues as new hA_3_ AR antagonists [160] (Figure 10).

The quinazoline-4-carboxamide (**QZ**) series, including 2-oxo- and 2-amino-substituted, maintain all key interactions evidenced for the **TQX** derivatives [146,149], i.e., π-π stacking interactions with both side chains of Phe168 (EL2) and Phe182 (TM5), two H-bonds with Gln167 and Asn250, and an H-bond with His95. The intramolecular H-bond between N3 of the quinazoline system and the NH of the 4-amide moiety preserved the planarity and similar steric properties to the **TQX** analogues. The **QZ** derivatives resulted in potent hA_3_ AR antagonists (Table 7), and exhibited very high selectivity for the hA_3_ AR. As observed in the **TQX** series, the 2-oxo-derivatives displayed higher hA_3_ AR affinity compared to the corresponding 2-amino analogues (compare **43**, **44** to **45**, **46**) likely due to a stronger H-bonding interaction of the 2-carbonyl group with His95, compared to the 2-amino group [141,142,146,149]. The para-OMe substituent on the phenyl moiety (R) was confirmed to be the most favorable (compounds **44** and **46**) among those evaluated (OMe, Me, Br). The presence of acyl groups on the 2-amino moiety (derivatives **47** and **48**) enhanced hA_3_ AR affinity, in comparison to the unsubstituted compound **45**.

The quinoline-4-carboxamide (**QN**) derivatives (Figure 10) exhibited no affinity for the hA_3_ AR because the lack of the intramolecular H-bond does not allow the system to adopt a planar conformation, thus losing some of the most important interactions with the receptor binding site. In the pyrimidine-4-carboxamide derivatives (**PYRM** series, Figure 10), the intramolecular H-bond allowed a simulation of the planar bicycle, therefore maintaining the π-π stacking interactions with both Phe168 and Phe182. Nevertheless, these derivatives exhibited no hA_3_ AR affinity, likely due to a shift in their positioning within the binding cleft which prevents the formation of H-bonding interactions with His59, Gln167, and Asn250.

#### 2.1.4. From **TQX** and **QZ** Series to the 2-Phenylphthalazin-1(2H)-one Derivatives

**Figure 11:**
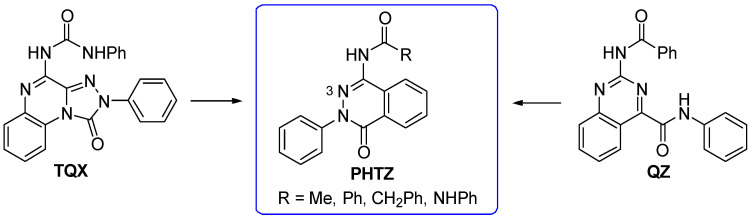
Design of the 2-phenylphthalazin-1(2H)-one derivatives as analogues of **TQX** and **QZ** series.

To continue pursuing a molecular simplification approach for the design of novel hA_3_ AR antagonists, 2-phenylphthalazin-1(2H)-one derivatives (**PHTZ**, Figure 11) [161], featuring amido and ureido moieties at the 4-position, were synthesized due to their structural similarity with both **TQX** [141] and **QZ** series [160].

Some of the **PHTZ** compounds displayed both high affinity and selectivity for hA_3_ AR (derivatives **49**–**54** Table 8); the most active compounds were the 4-arylureido-substituted bearing methoxy group(s) on the phenyl pendant (derivatives **51**–**53**), which displayed K_i_ values in the low nanomolar range, the best being derivative **53** (K_i_ = 0.776 nM).

The benzylureido derivative **54** also exhibited high hA_3_ AR affinity and selectivity. Surprisingly, acylamino substituents at position 4 were detrimental, resulting in weakly active (e.g., **49**), or completely inactive compounds, in contrast with the positive effect of these moieties in other series of hA_3_ AR antagonists, including the **TQX** and **QZ** series. Compounds **51**–**54** acted as potent antagonists by reversing NECA-mediated inhibition of cAMP levels in hA_3_-CHO cells, while were inactive in cAMP assay in hA_2B_-CHO cells. The new **PHTZ** derivatives were docked at the first published homology model of the hA_3_ AR based on the crystal structure of the A_2A_ AR in complex with the antagonist ZM241385 (PDB code: 3EML) [162]. Derivative **50** was accommodated into the binding cavity with the 4-phenylureido group oriented toward the EL region, while maintaining all the key interactions observed for the **TQX** and **QZ** derivatives. The compound forms three H-bonds with Asn250 (TM6) side chain, involving the N3 atom of the pthalazinone core and the two NH groups of the ureido moiety. Additionally, the **PHTZ** core engages in π-π-stacking interactions with Phe168 (EL2).

#### 2.1.5. From **TQX** Series to the 1,2,4-Triazolo[4,3-a]pyrazin-3-one Derivatives

**Figure 12:**
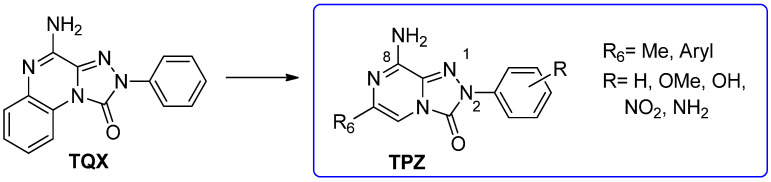
Design of the 1,2,4-triazolo[4,3-a]pyrazin-3-one series as simplified analogues of **TQX** series.

With the aim of identifying a novel bicyclic chemotype, the 2-aryl-8-amino-1,2,4-triazolo[4,3-a]pyrazin-3-one (**TPZ**) series was designed through structural simplification of the **TQX** series (Figure 12). Notably, the synthetic accessibility of the **TPZ** scaffold allowed the introduction of various substituents with different lipophilic and steric properties at the 6-position (Me, aryl) and on the 2-phenyl ring (OMe, OH, NO_2_, NH_2_) [163]. Moreover, replacement of the fused benzo ring of **TQZ** with a flexible 6-phenyl pendant might enhance both the molecular fit within the receptor site and the solubility of the compounds in the assay media.

All derivatives were totally inactive at the hA_2B_ AR, as they exhibited IC_50_ values > 30,000 nM in the cAMP assays. The 2,6-diphenyl-substituted derivative **56** displayed nanomolar affinity for hA_1_, A_2A_, and hA_3_ ARs, while its analogue 6-methyl-substituted **55** was significantly less active. The presence of a substituent on the 2-phenyl ring (R) enhanced affinity for the hA_1_ AR while reducing that for hA_2A_ and hA_3_ ARs (**57**, **58**) (Table 9).

To enhance hA_2A_ AR affinity and selectivity, various substituents were introduced on the 6-phenyl ring, while maintaining R = H. The interest in identifying new hA_2A_ AR antagonists was due to their significant therapeutic potential in various diseases, including cancer, neurodegenerative disorders, and neuropathic pain. The new 6-aryl-triazolopyrazines **59**–**62** featuring a methoxy or other small alkoxy residues, on the para position of the 6-phenyl ring, exhibited high affinity (K_i_ = 2.9–10.6 nM) and complete selectivity for the hA_2A_ AR subtype. Compound **59** exhibited an antagonistic behavior (cAMP assay) and neuroprotective effect against MPP^+^-induced neurotoxicity in SH-SY5Y cell cultures, an in vitro model of Parkinson’s disease.

Additional triazolopyrazine derivatives featured a phenyl ring at position 2 (R_2_) and different aryl groups (R = NO_2_, Br, Cl, substituted piperazine) at position 6 (R_6_) [164]. To improve compound solubility, heteroaryl rings were also introduced in R_6_, and a flexible benzyl chain was incorporated in R_2_ (Table 10). All these triazolopyrazines were inactive at the hA_2B_ AR. Several compounds possessed nanomolar affinity for both hA_1_ and hA_2A_ ARs, and different degrees of selectivity versus the hA_3_ AR. Among the 2-phenyl-substituted derivatives, **63** (R_6_ = 2-furyl) and **66**, **67** (R = 3-Cl and 4-Cl on the 6-aryl) were the most active at both A_1_ and A_2A_ ARs. The same dual profile was exhibited by the 2-benzyl derivatives **68**–**70**. Derivatives **64** and **65**, bearing, respectively, a para nitro and bromine on the 6-phenyl ring, displayed high affinity (K_i_ = 7.2 and 10.6 nM, respectively) and a complete selectivity for the hA_2A_ AR.

In cAMP assays, derivative **65** acted as an antagonist at the hA_2A_ AR whereas derivatives **66** and **70** behaved as dual hA_1_/hA_2A_ AR antagonists (**66**, **70**). The three compounds were tested for their ability to counteract β-amyloid peptide (Aβ)-induced toxicity in SH-SY5Y cell lines, since data in the literature reported that selective hA_2A_ AR antagonists mitigated cognitive impairments induced by β-amyloid peptide administration in various in vivo rodent models [119,165,166] and that dual hA_1_/hA_2A_ AR antagonists were neuroprotective against β-amyloid-induced toxicity in neuroblastoma cells treated with aluminum chloride [167]. Derivative **66** did not exhibit a protective effect on cell viability, while compounds **65** and **70** demonstrated neuroprotective activity, by counteracting the neurotoxicity induced by Aβ aggregates exposure.

Finally, 2-phenyl-substituted **TPZ** derivatives were decorated on the para position of the 6-phenyl ring with amide moieties bearing terminal basic rings (pyrrolidine, piperidine, substituted piperazines, and morpholine) (Table 11) [168]. All compounds **71**–**74** exhibited one-digit nanomolar affinity for the hA_2A_ AR and high selectivity versus hA_1_ and hA_3_ ARs and also the hA_2B_ AR, being totally inactive in the cAMP assays in hA_2B_ AR-CHO cells (IC_50_ > 10,000 nM).

**Figure 13:**
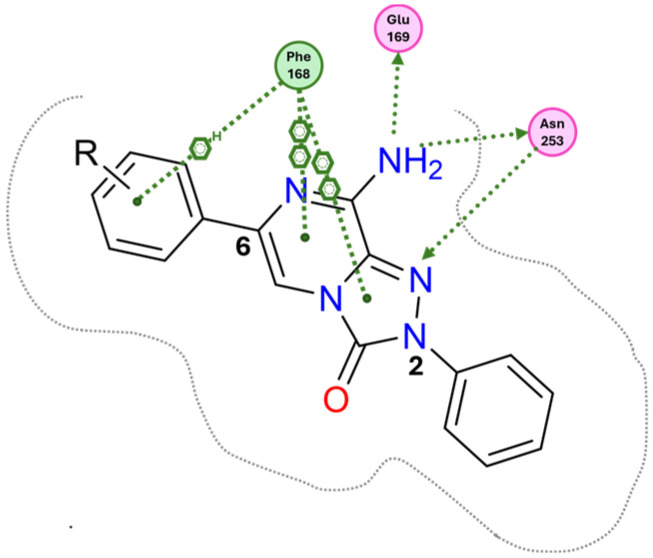
Schematic representation of the type one docking conformation of **TPZ** derivatives at the hA_2A_ AR.

Docking studies at the crystal structure of the hA_2A_ AR showed that steric hindrance of the substituents at the 2- and 6-positions of the **TPZ** bicyclic core influenced the ligand orientation within the binding cavity. Most of the triazolopyrazine derivatives (**55**–**62**, **64**–**67**) [163,164] preferentially bind to the receptor site with the 2-phenyl substituent positioned in the depth of the cavity, and the R_6_ group pointing toward the entrance of the binding site (type one conformation, Figure 13), mimicking the binding mode of **ZM241385** in the A_2A_ AR. The bicyclic core engages in π-π interaction with Phe168 (EL2) and the 8-NH_2_ group forms H-bonds with Asn253 (TM6) and Glu169 (EL2). Asn253 residue seems to interact also with the endocyclic N^1^ atom. The presence of a substituent on the 2-phenyl ring decreases A_2A_ AR affinity as it causes a slight shift of the molecule that reduces ability to establish some key interactions, such as those with Asn253 and Glu169.

Compounds bearing a heterocyclic moiety at position 6 and a phenyl ring at position 2 (e.g., derivative **63**) [164] can adopt two docking conformations: type one and type two. A slight preference is observed for type two in which the orientation is reversed: the smaller 6-substituent penetrates the binding cavity, and the bulkier 2-substituent points outward. Derivatives **64**–**67** preferentially adopt the type one conformation, whereas compounds **68**–**70**, bearing a benzyl group at position 2 and a heterocycle at position 6, favor the type two pose. This preference is driven by steric hindrance, positioning the bulkier 2-substituent toward the extracellular space.

Derivatives **71**–**74** assumed similar docking conformations to those observed for the lead compound **56**, presenting the bulky 6-aryl substituent oriented toward the extracellular environment. The lateral basic rings, which may carry a positive charge at pH = 7.4, were involved in polar interaction with the backbone carbonyl group of the nearby amino acids, as well as with the side chains of Ser67 and Ser6, at the entrance of the cavity. The carbon residue of the amide linker (X = NHCO) was capable of forming an H-bond with the OH group of Tyr271 (TM7). A similar interaction can also occur with the oxygen atom of the ether linker (X = O), but it is associated with lower binding energy.

##### 1,2,4-Triazolo[4,3-a]pyrazin-3-ones as Multi-Functional A_2A_ AR Antagonists

Traditional pharmacological approaches rely on the use of drugs that are as selective as possible for a specific target, with the aim of minimizing side effects arising from interactions with off-target biomolecules. However, it is well known that certain disorders, including neurodegenerative diseases, neuropathic pain, and cancer, involve complex pathways associated with the dysfunction of multiple biological targets. In this context, traditional therapies have revealed the limitations of the one-drug-one-target paradigm. Thus, in the recent years, the multifunctional approach has gained increasing relevance in the treatment of the aforementioned diseases. This approach can be pursued by two different strategies: (1) a combination of monofunctional drugs, each active on a specific target and administrated either in association or within the same formulation; or (2) the use of a single multifunctional compound capable of modulating multiple biological targets [169,170].

1. Antioxidant-hybridized 1,2,4-triazolo[4,3-a]pyrazin-3-ones

**Figure 14:**
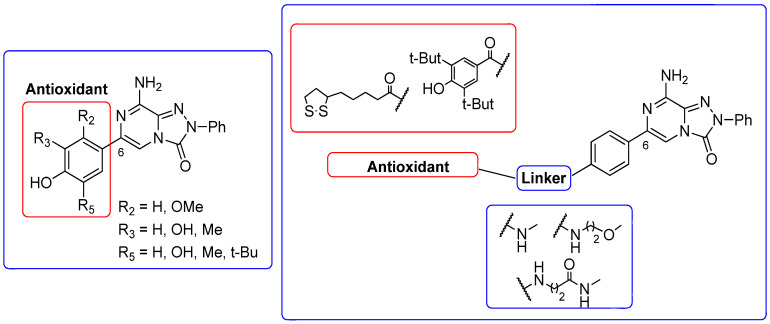
Antioxidant-based triazolopyrazin-3-one derivatives.

A_2A_ AR signaling plays a key role in modulating neuroinflammatory pathways, which are involved in various disorders, including neuropathic pain. Although the role of the A_2A_ AR in pain is still a matter of debate, since both pro-nociceptive and antinociceptive effects have been reported, A_2A_ AR blockade appears to confer protection [35]. Neuropathic pain is a condition characterized by a complex pathophysiology involving both central and peripheral mechanisms [35]. Although the molecular basis is not fully understood, oxidative stress might contribute to its development prompting growing interest in antioxidants as potential therapeutic agents [171,172,173]. Therefore, we focused on the development of dual-acting compounds exhibiting both A_2A_ AR antagonism and antioxidant activity, as they may offer enhanced therapeutic efficacy against neuropathic pain. To this end, two sets of novel triazolopyrazine derivatives were designed and synthesized, incorporating various antioxidant moieties (Figure 14) [174].

The new compounds exhibited nanomolar activity at the hA_2A_ AR and different degrees of selectivity versus hA_1_ and hA_3_ ARs (Table 12), while they were inactive in the cAMP assay in hA_2B_-CHO cells.

In the first set, phenolic residues at the 6-position yielded derivatives that showed nanomolar affinity for both hA_1_ and hA_2A_ ARs and high selectivity versus hA_3_ AR (derivatives **75**, **76**). Notably, the compound featuring the 3-terbut-4-phenol ring (derivative **77**) exhibited high hA_2A_ AR affinity (K_i_ = 8.5 nM) and high selectivity versus all the other AR subtypes.

The second set of triazolopyrazines contained α-lipoyl and 3,5-di-tert-butyl-4-hydroxybenzoyl residues as antioxidant portions which were appended via either an ether linkage or an amide bond to the para position of the 6-phenyl ring (Figure 14). The choice of this position was guided by molecular docking studies at the hA_2A_ AR, which indicated that bulky substituents on the 6-phenyl ring favor a binding pose in which this moiety is oriented toward the extracellular side of the receptor. As expected, these new derivatives showed nanomolar A_2A_ AR affinity and high selectivity. The most potent compounds were the lipoyl derivatives **78** and **79** (K_i_ = 2.4 and 36.4 nM, respectively) and compound **80**, featuring the 3,5-di-tert-butyl-4-hydroxyphenyl residue.

Compounds **77**, **78**, and **80** were evaluated in vitro for their neuroprotective activity against oxaliplatin-induced toxicity in microglial cells. To assess the specific contribution of A_2A_ AR antagonism, compound **81**, lacking the antioxidant moiety, was also tested. All compounds exhibited protective effects, with compounds **78** and **80** additionally reducing ROS levels, suggesting a direct antioxidant mechanism. Compounds **78**, **80**, and **81** were further evaluated in a mouse model of oxaliplatin-induced neuropathic pain, where they demonstrated efficacy in alleviating pain-related symptoms. The lipoyl derivative **78** emerged as the most potent, completely reversing oxaliplatin-induced pain. The efficacy of compound **81**, despite lacking an antioxidant moiety, underscores the contribution of A_2A_ AR antagonism to the reduction of oxaliplatin-induced neurotoxicity, both in vitro and in vivo.

2. First-in-Class Multi-Target Adenosine A_2A_ AR Antagonists–Carbonic Anhydrase IX and XII Inhibitors

Another study employing a multi-target approach within the triazolopyrazine series led to the development of the first-in-class multi-target agents acting as both hA_2A_ AR antagonists and human carbonic anhydrase (hCA) IX and XII inhibitors, representing promising new candidates for anticancer therapy [175]. In recent years, the A_2A_ AR and hCAs IX and XII have emerged as promising targets for anticancer therapy. A_2A_ AR is frequently upregulated in the tumor microenvironment, where its activation suppresses immune cell function and promotes tumor cell proliferation, thereby facilitating immune evasion, tumor progression, and metastasis [105,106,107,108,109,110,176,177].

CAs are a family of ubiquitous metalloenzymes characterized by the presence of a zinc ion in their catalytic site. Although they exhibit distinct catalytic efficiencies and tissue-specific expression patterns, their most prominent physiological roles include the regulation of pH homeostasis and the facilitation of ion transport [178,179,180]. In particular, the membrane-bound isoforms hCAs IX and XII are markedly upregulated in hypoxic tumors. Their overexpression contributes to the maintenance of an alkaline intracellular pH, preventing acidification within tumor cells while simultaneously promoting extracellular acidosis. These pH imbalances are known to favor the initiation and progression of solid tumors. Consequently, tumor-associated hCAs IX and XII represent promising therapeutic targets to counteract tumor growth and metastasis [179].

**Figure 15:**
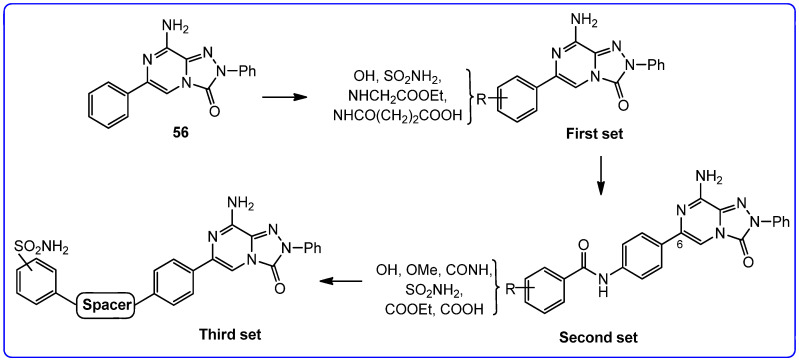
Design of triazolopyrazines as multitarget human A_2A_ AR antagonists–carbonic anhydrase IX and XII inhibitors.

On this basis, our efforts were devoted toward the development of dual-acting compounds capable of simultaneously blocking both hA_2A_ AR and hCAs IX and XII, to achieve a synergistic anticancer effect. Thus, the potent A_2A_ AR antagonist triazolopyrazine **56** was decorated with various zinc-binding groups (ZBGs) (Figure 15).

The new compounds exhibited binding activity at hA_1_, hA_2A_, and hA_3_ ARs at concentrations ranging from micromolar to nanomolar, while they were inactive in the cAMP assay in hA_2B_-CHO cells (Table 13).

In the first set, simple substituents able to coordinate the zinc ion either directly or via a water molecule (-OH, –COOH, –CONHOH, and –SO_2_NH_2_), were introduced on the 6-phenyl ring of compound **56**. Among them, the sulfonamide moiety proved to be the most effective, yielding derivative **82**, which showed some affinity for the hA_2A_ AR and good inhibitory activity against hCA IX/XII isoforms. In contrast, compounds bearing a hydroxy group exhibited high hA_2A_ AR affinity but weak inhibition of hCA isoforms.

In the second set, the ZBGs were introduced on a lateral phenyl ring, attached to the 6-phenyl group through an amide linker. Due to the presence of the longer tail, several compounds exhibited nanomolar affinity and enhanced selectivity for the hA_2A_ AR, but limited or no inhibitory activity against hCA isoforms. The only notable exception was compound **83**, which demonstrated potent dual-target activity, confirming benzenesulfonamide as the most effective ZBG for simultaneously targeting hA_2A_ AR and hCA XII (K_i_ = 6.4 and 6 nM, respectively, Table 13).

In the third set, the benzenesulfonamide moiety was connected to the 6-phenyl ring via spacers of varying length and flexibility. The new triazolopyrazines showed nanomolar affinity for hA_2A_ AR and inhibited hCAs with different degrees of selectivity. Among them, derivatives **84** exhibited the best profile, as it showed good affinity for hA_2A_ AR (K_i_ = 108 nM) and inhibited both the tumor-associated hCA IX and XII isozymes at nanomolar concentration (K_i_ = 5.0 and 27.0 nM, respectively).

Docking studies showed that these compounds bind to the hA_2A_ AR similarly to the previously reported triazolopyrazines (type one conformation). In hCA II, IX, and XII isoforms, the benzenesulfonamide moiety coordinated the catalytic Zn(II) ion and established two hydrogen bonds with Thr199. An increased distance between the sulfonamide and the scaffold facilitated better accommodation within the enzyme active sites, with spacer length and flexibility critically influencing binding efficiency.

### 2.2. 2-Arylpyrazolo[3,4-c]quinolone Derivatives and Their Simplified Analogs

#### 2.2.1. 2-Arylpyrazolo[3,4-c]quinoline Derivatives

**Figure 16:**
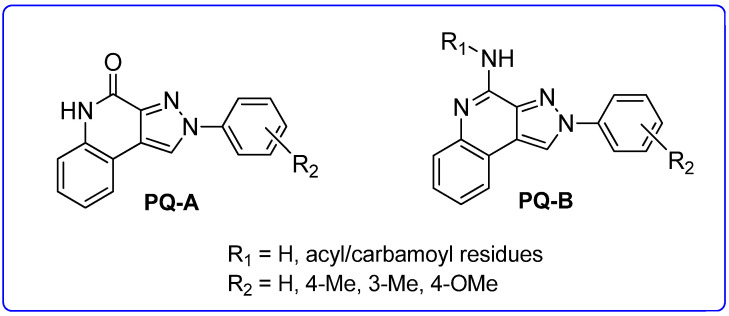
2-Arylpyrazolo[3,4-c]quinoline derivatives **PQ-A** and **PQ-B**.

In addition to the **TQX** derivatives (Section 2.1), compounds with 2-arylpyrazolo[3,4-c]quinoline structure, differently substituted at position 4 to give, respectively, the 4-one (**PQ-A**) and 4-amino (**PQ-B**) series, were deeply investigated (Figure 16). Indeed, the pyrazolo[3,4-c]quinoline core proved to be a versatile scaffold for the development of potent and selective hA_3_ AR antagonists.

##### Pyrazolo[3,4-c]quinolin-4-one Derivatives

In the **PQ-A** series, the 2-phenyl substitution pattern strongly influenced AR binding (Table 14). While all **PQ-A** compounds did not bind to the hA_2A_ receptor, they exhibited good hA_1_ and hA_3_ binding affinity, but limited hA_3_ versus hA_1_ selectivity. The 4-methoxy substitution on the 2-phenyl ring (**87**) afforded the best selectivity (hA_3_/hA_1_ ratio = 55), followed by 4-methyl (**86**, hA_3_/hA_1_ ratio = 9). Substitution of the 2-phenyl moiety with a benzyl one (**88**) preserved hA_3_ AR affinity but eliminated hA_1_ binding, indicating the hA_3_ pocket can accommodate bulkier groups. In contrast, replacing the 2-phenyl moiety with the smaller 2-methyl group (**89**) abolished binding activity, highlighting the need for more hindering, lipophilic substituents at this position [181,182].

Ligand-based homology modeling (LBHM) supported these findings. Ligand recognition occurred in the upper region of the TM bundle, with the pyrazoloquinoline core near TMs 3, 5, 6, and 7. Key hydrogen bonds involved Thr94, His95, and Ser247 interacting with the 4-carbonyl group. Additionally, the 4-methoxy group on the 2-phenyl moiety (**87**) likely engaged in a weak hydrogen bond with Ser165 in the second extracellular loop (EL2) contributing to enhanced affinity and selectivity [154].

To improve affinity for A_1_ and/or A_2A_ ARs, **PQ-A** derivatives bearing bulky, lipophilic groups on the benzo-fused moiety were synthesized, as suggested by the literature data [13,142,183]. However, all compounds proved inactive at the targeted receptors [182].

##### Pyrazolo[3,4-c]quinolin-4-amino Derivatives

Several structural modifications on the **PQ-B** series (Figure 14) were carried out to explore SARs [154,181,182,184]. Accordingly, 4-amino-, 4-amido-, and 4-benzylureido-pyrazoloquinoline derivatives were synthesized (**90**–**106**, Table 15) [154,181]. The amino derivatives (R_1_ = H, compound **90**) generally showed good hA_1_, hA_2A_, and hA_3_ AR affinities. In contrast, 4-acylamino, 4-benzylureido, and 4-diacylamino compounds (**91**–**102**) displayed significantly higher affinity and selectivity for the hA_3_ receptor subtype.

Docking studies showed that **PQ-B** compounds interact with the upper part of the binding pocket (TMs 3, 5, 6, 7) with the 4-substituent oriented toward the intracellular environment and the 2-phenyl ring positioned near to TMs 3, 6, and 7. Polar amino acid residues such as Thr94, His95, and especially Ser247 in the hA_3_ AR are key for receptor selectivity, forming hydrogen bonds with the 4-amido/ureido groups. In particular, the hydroxyl group of Ser247 engaged in hydrogen-bonding interaction with the carbonyl oxygen of the 4-amido/ureido moieties. Ser247 was absent in hA_1_ and hA_2_ receptors, where a bulkier histidine residue prevents access for large 4-acylamino and 4-benzylureido substituents explaining the low affinity of compounds **91**–**102** for those receptor subtypes. In general, the hA_3_ AR affinity of 4-N-acylated derivatives increased with the bulkiness of the N4 substituent (compare **91**, **92** to **93**–**98**) that accommodated in the hydrophobic region delimitated by five nonpolar amino acids (Ile98, Ile186, Leu190, Phe239, and Leu244). Hydrophobic substituents (Me, OMe) on the 2-phenyl ring did not influence binding affinity. This was particularly evident for 4-N-acylated derivatives. As the size of the N4 substituent increases, the position of the 2-phenyl group moves away from EL2 leading to the loss of the hydrogen-bonding interaction between the methoxy group and the Ser165 residue [154].

Further SAR studies introduced various heteroaroyl or aroyl groups at the 4-amino position [184]. Compounds bearing the 2-furyl or 4-pyridyl ring on the 4-amido group (**103**, **104**, Table 15) showed nanomolar hA_3_ AR affinity and high selectivity. Substituents on the 4-benzoylamino group produced variable effects: methyl groups preserved activity (**105**), while trifluoromethyl groups reduced affinity. Finally, introducing bulky and lipophilic groups at position 6 of the benzo-fused ring (**106**) shifted affinity towards the hA_1_ receptor subtype [182].

Compounds **97** and **102** showed potent inhibition of NECA-modulated adenylyl cyclase activity in CHO cells, with EC_50_ values of 3.8 nM and 11.2 nM, respectively. As expected, their affinity at the rA_3_ AR was lower than that at the hA_3_ AR.

In a rat model of cerebral ischemia, both compounds protected against OGD-induced neuronal damage and delayed or prevented anoxic depolarization, confirming their neuroprotective effects. As mentioned for derivatives **27** and **39**, these results are consistent with those previously observed in the same brain region using other potent hA_3_ AR antagonists, exhibiting low binding affinity at the rA_3_ AR [150,159].

#### 2.2.2. From **PQ** series to Pyrazolo[4,3-d]pyrimidine Compounds

**Figure 17:**
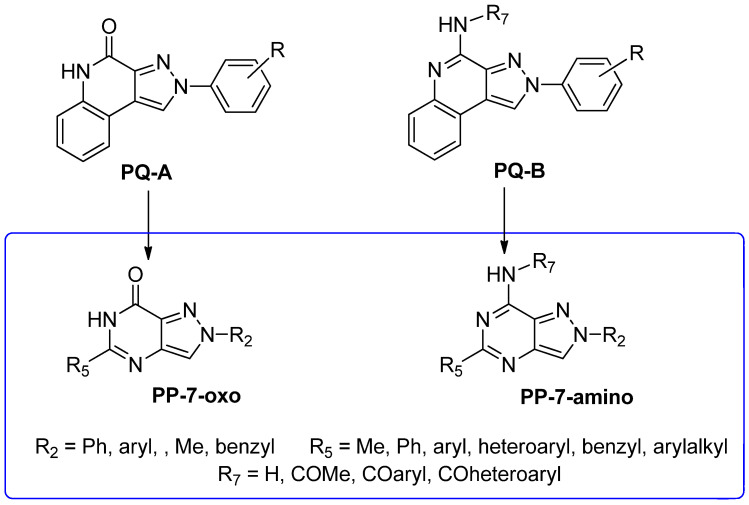
Design of pyrazolo[4,3-d]pyrimidine.

To develop new hA_3_ AR-targeting compounds with improved physicochemical properties, a molecular simplification of the **PQ-A** and **PQ-B** scaffolds led to the 2-arylpyrazolo[4,3-d]pyrimidin-7-one (**PP-7-oxo**) and -7-amino (**PP-7-amino**) series, respectively (Figure 17) [162,185,186,187,188,189].

This strategy improved hA_3_ versus hA_1_ selectivity, while maintaining or enhancing affinity for the target receptor. To explore SARs, substituents with varying steric bulk, flexibility, and lipophilicity (Figure 17) were introduced at the 5- and 2-positions of the bicyclic scaffold.

##### Pyrazolo[4,3-d]pyrimidin-7-one Derivatives

Notably, **PP-7-oxo** derivatives (**107**–**113**, Table 16) showed nanomolar affinity and high selectivity at the hA_3_ AR. Introduction of small R_5_ substituents such as methyl (**108**) was preferred over bulkier groups like phenyl (**109**) or benzyl (**110**), likely due to a better accommodation within a lipophilic receptor pocket of limited size. Among the modifications on the 2-phenyl ring, the 4-OMe group (**111**) conferred the highest hA_3_ AR affinity (K_i_ = 1.2 nM, Table 16), outperforming other substitutions such as 3-Me and 4-Me (**112**, **113**) [162,185]. To depict the binding mode of these new potent and selective hA_3_ AR antagonists, a new homology model of the hA_3_ AR using the crystal structure of the hA_2A_ receptor (PDB code: 3EML) as a template was built [162]. Molecular docking studies indicated that the pyrazolo[4,3-d]pyrimidin-7-one scaffold is embedded within TMs 3, 5, 6, and 7, engaging in aromatic π-π stacking interactions with Phe168. The 2-phenyl ring is oriented toward EL2, while the R_5_ substituent is directed toward the intracellular environment. Small R_5_ substituents enabled the formation of two stabilizing hydrogen-bonds—one involving the carbonyl group at position 7 and the other the nitrogen atom of the pyrazole ring—both interacting with the amide moiety of Asn250. In contrast, derivatives bearing bulkier R_5_ groups (phenyl (**109**), benzyl (**110**)) adopted an altered binding orientation, redirecting the 2-phenyl ring away from EL2 and positioning R_5_ toward TM2. This rearrangement disrupted the π-π stacking interaction with Phe168 and eliminated one of the hydrogen bonds with Asn250 potentially accounting for the absent or diminished hA_3_ AR binding affinity of compounds **109** and **110** [162].

##### Pyrazolo[4,3-d]pyrimidin-7-amino Derivatives

Substitution of the 7-oxo group of the pyrazolo[4,3-d]pyrimidine scaffold with an amino function yielded the **PP-7-amino** series (**114**–**118**, Table 17).

The free 7-amino function conferred good affinity for the hA_1_ and hA_2A_ ARs (compounds **114**, **116**, and **117**), while it affected hA_3_ affinity based on R_5_ substituents [185,186]. Substituents with varying steric bulk, flexibility, and lipophilicity (R_5_ = Me, Ph, C_6_H_4_-4-OMe, 2-thienyl) were well tolerated at hA_1_ and hA_2A_ subtypes, while for hA_3_ affinity heteroaryl or aryl substituents (**117**, **118**) were necessary. Introduction of a 4-OMe group on the 2-phenyl ring did not consistently enhance hA_3_ AR affinity and selectivity.

To optimize hA_3_ AR affinity, acyl groups were added to the 7-amino moiety (**119**–**127**, Table 17) [185,186]. These 7-amido derivatives displayed nanomolar affinity for the hA_3_ AR, with minimal activity at the other AR subtypes, the most active being **125** (K_i_ = 0.027 nM). Even compounds with two acyl groups (**129**, Table 17) retained high affinity, suggesting the existence of a spacious receptor pocket. Molecular modeling showed that ligand recognition took place in the upper portion of the transmembrane domain, stabilized by hydrogen bonds with Asn250 and π-π stacking interaction with Phe168. Due to this specific binding pose, the R_7_ group was positioned facing outward from the binding cleft.

Compounds **119** (K_i_ = 5.6 nM), **121** (K_i_ = 18 nM), **122** (K_i_ = 18 nM), and **123** (K_i_ = 24 nM) improved cell viability in rat astrocyte cultures exposed to oxaliplatin with **123** being the most effective [186]. Importantly, compound **123** did not interfere with the antineoplastic activity of oxaliplatin in HT-29 cancer cells, indicating distinct mechanisms of oxaliplatin toxicity in normal neurons versus tumor cells. However, these compounds lacked affinity for the rat A_3_ AR, confirming known species differences between rat and human [190]. Therefore, these results support an A_3_ receptor-independent mechanism.

Introduction of arylalkyl and heteroaryl substituents at positions 2 and 5 of the **PP-7-amino** scaffold led to potent hA_2A_ or balanced hA_2A_/hA_1_ antagonists (**129**–**134**, Table 18) [187,188,189]. The free 7-amino group was crucial for hA_1_ and hA_2A_ selectivity, while the 2- and 5-substituents modulated receptor preference. Specifically, a phenyl group at position 2 and an arylalkyl chain at position 5 (**129**, **130**) yielded dual-acting hA_1_/hA_2A_ antagonists with higher affinity toward the A_1_ AR subtype. In contrast, a heteroaryl moiety at position 5 (e.g., 2-furyl or 2-(5-methylfuryl)) and a (substituted) benzyl group at position 2 (**131**–**134**) favored A_2A_ AR selectivity.

#### 2.2.3. Imidazo[1,2-a]pyrazin-8-amino Derivatives

Building on the success of **PP**-based hA_3_ AR antagonists, a series of 2-aryl-imidazo[1,2-a]pyrazin-8-amino derivatives (**IP** series, Table 19) was developed [191]. These compounds also possess features typical of fluorescent heteroaromatic systems [192,193]. The aim was to optimize both hA_3_ AR affinity and fluorescence emission properties. In particular, the N^8^-(hetero)arylcarboxamido-substituted **IP** compounds, either with or without a 6-phenyl group (**135**–**139**, Table 19) showed good hA_3_ AR affinity and selectivity, but lacked activity at the rat A_3_ AR, confirming species-specific differences. Some derivatives exhibited moderate fluorescence, though not sufficient for use as biological probes.

Compound **139** was selected for further investigation. In a rat model of cerebral ischemia, it significantly restored impaired neurotransmission, likely through a mechanism independent of A_3_ receptor antagonism and possibly involving an intracellular target.

#### 2.2.4. Thiazolo[5,4-d]pyrimidine Derivatives

##### Thiazolo[5,4-d]pyrimidin-7-one Derivatives

**Figure 18:**
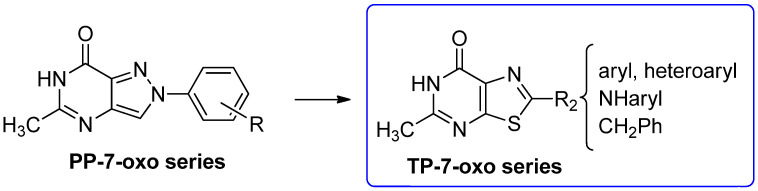
Design of thiazolo[5,4-d]pyrimidin-7-one derivatives.

Starting from 2-arylpyrazolo[4,3-d]pyrimidin-7-one (**PP-7-oxo** series) derivatives [162], a novel class of thiazolo[5,4-d]pyrimidin-7-one (**TP-7-oxo** series) compounds was rationally designed (Figure 18) [194].

The thiazolopyrimidine scaffold maintains key structural features, such as the 7-oxo function and the nitrogen at position 1, that are important for hA_3_ receptor binding, and incorporates a methyl group at position 5, in line with previous findings in the lead series for enhancing hA_3_ AR antagonist activity [162].

SAR studies showed that a 2-phenyl group bearing a para-hydrophobic substituent (e.g., Cl, OMe, Me, **140**–**142**) enhanced hA_3_ AR affinity, while a hydrophilic group (e.g., OH, **143**) reduced it (Table 20). Increasing the distance between the 2-phenyl and the bicyclic core using linkers (e.g., methylene in **144** or the amino group in **145**) also led to decreased affinity.

##### Thiazolo[5,4-d]pyrimidin-7-amino Derivatives

**Figure 19:**
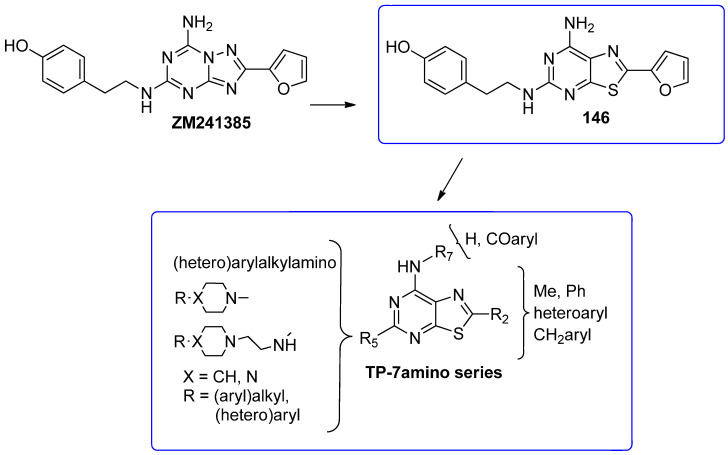
Design of new **TP**-based hA_2A_ AR antagonists/inverse agonists.

The thiazolo-pyrimidine (**TP**) core resulted a versatile scaffold for the development of hA_2A_ AR antagonists. Starting from the hA_2A_ AR inverse agonist **ZM241385** (Figure 19) [156], a bioisosteric replacement of its triazolotriazine core with a thiazolopyrimidine led to compound **146** [195].

While **146** (Table 21) showed affinity for all hARs, it lacked selectivity for hA_2A_ over hA_1_ AR. To improve selectivity, many other **TP** derivatives were synthesized, taking advantage of the ease of modifying the TP core at positions 2, 5, and 7 (Figure 19).

Keeping the 2-furane ring and the 7-amino group constant, substitutions at position 5 were explored [195,196,197,198]. Compounds **147**–**152** (Table 21) [195,196], featuring a (hetero)arylalkylamino chain at position 5, showed two affinity values (KH in the femtomolar, KL in the nanomolar range) for the hA_2A_ AR and potent inverse agonist activity, significantly inhibiting basal cAMP accumulation (IC_50_ in the picomolar range). Despite moderate to good/high affinities for hA_1_, hA_3_, and hA_2B_ ARs, compounds **147**–**152** are among the most potent and selective hA_2A_ AR inverse agonists reported, leading to their patenting [199].

Given the known role of A_2A_ AR signaling in pain modulation [200,201], compounds **147** and **148** (Table 21) were tested in vivo alongside **ZM241385** and morphine [195]. In both the writhing and hot water tail immersion tests, they showed anti-nociceptive effects comparable or superior to morphine and greater than those of **ZM241385**.

Additionally, **147** was evaluated for its anti-cancer potential [202]. In A375 (melanoma), A549 (lung), and MRMT-1 (breast) cancer cell lines, selective A_2A_ AR activation promoted tumour cell proliferation through ERK1/2, JNK1/2, and AKT pathways. These effects were reversed by both **ZM241385** and compound **147**, supporting the role of A_2A_ AR in tumorigenesis and highlighting **147** as a promising anticancer agent.

To further investigate the role of substituents at position 5 in hA_2A_ receptor binding, 7-amino-2-(furan-2-yl)thiazolopyrimidines bearing a piperazine chain at position 5 were synthesized (compounds **153**, **154**, Table 21). These compounds showed potent inverse agonist activity and good selectivity toward the hA_2A_ AR, whether the piperazine was directly attached to the bicyclic core (**153**) or linked via an ethylamino spacer (**154**) [197]. Additionally, bioinformatics prediction revealed that they possessed good drug-likeness profiles [197]. Similar results were achieved when an aryl or heteroaryl group was directly attached at position 5 of the bicyclic scaffold (**155**, **156**), with compound **156**, featuring furan-2-yl groups at both positions 2 and 5, exhibiting the most favorable combination of hA_2A_ AR affinity and selectivity [203].

To enhance affinity and selectivity toward the hA_3_ AR subtype, various acyl groups were introduced on the 7-amino function. This led to compound **157** (Table 21), which showed high affinity and selectivity for the hA_3_ AR. As expected, this modification reduced hA_2A_ binding activity [203] confirming that a free 7-amino group is crucial for effective anchoring of ligands at the hA_2A_ AR subtype—a trend observed in other classes of similar AR antagonists [186,204,205].

Further SAR studies involved simultaneous modifications at positions 2 and 5, keeping the 7-amino group free. Replacing the 2-furan-2-yl group in compound **147** with phenyl, pyrazin-2-yl, methyl, or 2-thienyl group led to compounds **160**–**163** (Table 22), which displayed dual A_1_ AR antagonism/A_2A_ AR inverse agonism [196,198].

Additional dual-acting ligands (**164**, **165**, Table 22) were obtained by coupling a 2-phenyl group with (hetero)aryl substituents at position 5 [203]. Similarly, potent hA_1_/A_2A_ AR dual antagonists (**166**, **167**, Table 22) were obtained by combining 5-aryl or heteroaryl groups with 2-unsubstituted or ortho-substituted benzyl moieties [206].

Adenosine signaling has emerged as a promising target for depression treatment [207,208]. Caffeine, a weak non-selective A_1_ and A_2A_ AR antagonist, has shown behavioral effects in classical animal depression models [209]. Based on these findings, compound **166** (hA_1_ K_i_ = 1.9 nM; hA_2A_ K_i_ = 0.06 nM, Table 22) was evaluated for antidepressant-like activity. In the forced swim test (FST) and tail suspension test (TST), it showed efficacy comparable to amitriptyline. Additionally, in the sucrose preference test, compound **166** demonstrated significant anti-anhedonic effects also matching those of amitriptyline [206].

Docking studies using the high-resolution crystal structure of the hA_2A_ AR-**ZM241385** complex revealed that **TP** ligands align similarly to **ZM241385**, preserving key interactions: a double hydrogen bond with Asn253 (via N1 and the 7-amino group), polar contact with Glu169, π-π stacking with Phe168, and hydrophobic interaction with Leu249. The 2-substituent penetrates deep into the receptor cavity near TM3, TM5, and TM6, while the 5-substituent, oriented toward the extracellular space, can exhibit diverse steric and electronic properties and may be exploited for the design of multi-target agents.

Recent studies indicate that simultaneous inhibition of adenosine biosynthesis and A_2A_ AR activity can suppress tumor growth [210]. Based on this, new TP derivatives were developed to co-target CD73, the main enzyme in adenosine biosynthesis, and A_2A_ AR [211]. These multi-target compounds incorporate a 7-amino-2-(furan-2-yl)thiazolo[5,4-d]pyrimidine core with a 5-substituent linked to a benzenesulfonamide group, typical of CD73 inhibitors [212]. While most of these compounds showed strong inverse agonist activity at hA_2A_ AR (**158** Table 21), their CD73 inhibition was weak [211]. However, compound **159** (Table 21) emerged as an intriguing dual hA_2A_/hA_2B_ AR inverse agonist/antagonist with high selectivity over the hA_1_ and hA_3_ AR subtypes. Given the A_2B_ AR role in promoting tumor growth [213], compounds able to block A_2B_ ARs, or both A_2A_ and A_2B_ ARs, like **159**, hold promise as anticancer agents.

## 3. Conclusions

**Figure 20:**
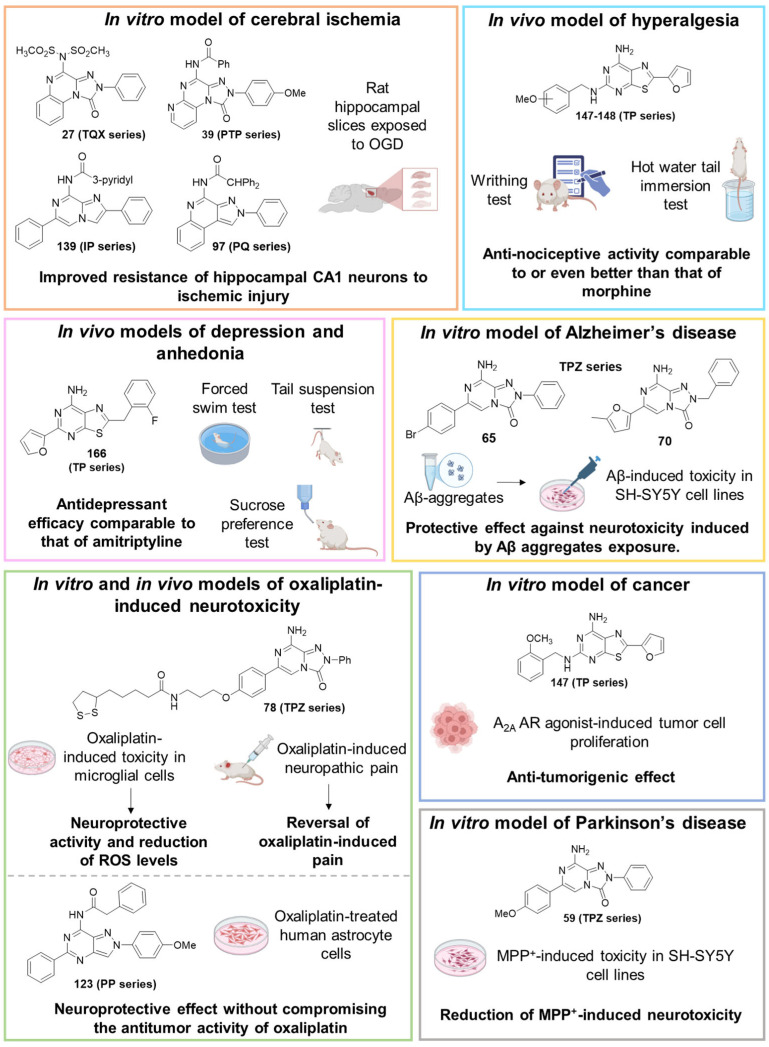
Summary of the pharmacological activity of selected AR antagonists in in vitro and in vivo models.

This review summarizes some research conducted by our group which has led to the discovery of some of the most potent and selective antagonists for the hA_2A_ and hA_3_ AR subtypes reported to date, characterized by diverse molecular chemotypes. Preliminary pharmacological investigations carried out with selected hA_2A_ AR and hA_3_ AR antagonists are briefly summarized in Figure 20.

The structural simplification from tricyclic to bicyclic scaffolds was partly aimed at addressing the frequently limited solubility of the compounds in assay media, which often hindered both in vitro and in vivo testing. This strategy proved successful, including in terms of AR affinity, particularly for the thiazolopyrimidine series, which yielded extremely potent hA_2A_ AR antagonists as well as dual hA_2A_/hA_2B_ AR antagonists with predicted favorable drug-like properties. Ongoing studies of this series aim to identify selective hA_2B_ AR antagonists as growing evidence highlights the significant therapeutic potential of compounds blocking the hA_2B_ AR. This is due to their ability to modulate immune responses, inhibit tumor growth, and regulate inflammatory signaling pathways. Antagonists of the hA_2B_ AR warrant further investigation also in neuroinflammatory diseases, such as cerebral ischemia and multiple sclerosis, in which they have demonstrated neuroprotective effect.

Our research has also led to the development of several highly potent and selective hA_3_ AR antagonists. However, the lack of affinity for the rat A_3_ receptor has limited their pharmacological characterization in both in vitro and in vivo models. To date, only a limited number of A_3_ AR antagonists have been shown to retain activity across both human and rodent receptors. Therefore, the discovery of pan-species A_3_ AR antagonists is highly desirable to facilitate more robust and predictive preclinical studies and to advance the development of A_3_ AR-targeted therapies. This need is particularly relevant given that A_3_ AR antagonists have demonstrated antitumor efficacy in preclinical models. Moreover, further studies are essential to unravel the dual and context-dependent role of A_3_ AR, promoting either cell proliferation or cell death.

Our research has also contributed to the development of multifunctional compounds as innovative potential drugs, e.g., dual-acting antioxidant–hA_2A_ AR antagonists as neuroprotective agents and human A_2A_ AR antagonists–carbonic anhydrase inhibitors, as potential antitumor agents. These multifunctional strategies may offer a more comprehensive and effective approach to address the multifactorial nature of complex disorders such as tumors and neurodegenerative diseases.

To conclude, we believe that the findings presented herein will be of interest to researchers working in the field of adenosine. The diversity of chemotypes and the therapeutic potential of AR antagonists may pave the way for future studies aimed at identifying novel compounds for the treatment of neuroinflammatory, neurological, and oncological diseases.

## Figures and Tables

**Table 1:** Binding affinity (K_i_) of **TQX-A** derivatives at bovine (b) A_1_ and A_2A_ ARs, and at human (h) A_1_ and A_3_ ARs ^a^.

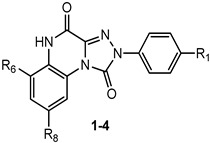							
				K_i_ (nM)			
	R_1_	R_6_	R_8_	bA_1_	hA_1_^b^	bA_2A_	hA_3_
**1**	H	H	H	515 ± 43	n.t.	>20,000	80 ± 6.3
**2**	OMe	H	H	934 ± 85	>20,000	>20,000	16 ± 1.2
**3**	NO_2_	H	H	>20,000	>20,000	>20,000	0.6 ± 0.03
**4**	OMe	NO_2_	H	>20,000	>20,000	>20,000	4.7 ± 0.52

^a^ Not tested at the hA_2B_ AR. ^b^ n.t. = not tested.

**Table 2:** Binding affinity (K_i_) of **TQX-B** derivatives at bovine (b) A_1_ and A_2A_ ARs, and at hA_1_ and hA_3_ ARs ^a^.

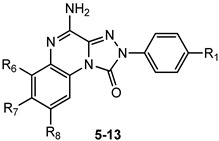								
					K_i_ (nM)			
	R_1_	R_6_	R_7_	R_8_	bA_1_	hA_1_^b^	bA_2A_	hA_3_
**5**	H	H	H	H	11.0 ± 0.83	n.t.	49.0 ± 3.7	490 ± 41
**6**	OMe	H	H	H	312 ± 27	69 ± 5.2	376 ± 30	45 ± 1.2
**7**	NO_2_	H	H	H	>20,000	n.t.	>20,000	28%
**8**	H	NO_2_	H	H	82 ± 7.4	n.t.	75.8 ± 6.9	4.75 ± 0.3
**9**	OMe	NO_2_	H	H	>20,000	>20,000	>20,000	47 ± 3.9
**10**	OMe	NH_2_	H	H	>20,000	186 ± 11.3	1049 ± 98.6	22 ± 1.9
**11**	H	H	Cl	H	47.8 ± 3.8	n.t.	113 ± 10	10.2 ± 0.9
**12**	H	H	H	Cl	17.1 ± 1	n.t.	102 ± 9.3	11 ± 1
**13**	H	NO_2_	H	Cl	0.2 ± 0.01	n.t.	256 ± 21	0.112 ± 9.3

^a^ Not tested at the hA_2B_ AR. ^b^ n.t. = not tested.

**Table 3:** Binding affinity (K_i_) of **TQX-B** derivatives at bA_1_ and bA_2A_ ARs, and at hA_1_ and hA_3_ ARs.

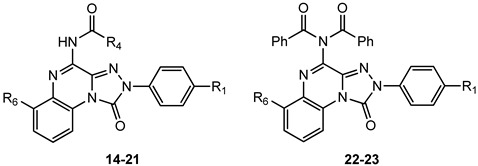								
				K_i_ (nM)				
	R_4_	R_1_	R_6_	hA_1_	hA_2A_	bA_1_	bA_2A_	hA_3_
**14**	Me	H	H	2000 ± 140	>10,000	4.3 ± 0.38	>20,000	2.0 ± 0.13
**15**	Ph	H	H	87.8 ± 6.30	88.2 ± 5.8	89.6 ± 7.2	>20,000	1.47 ± 0.11
**16**	CHPh_2_	H	H	18.8 ± 1.2	>10,000	10.2 ± 1.6	1160 ± 97.4	0.81 ± 0.03
**17**	Ph	OMe	H	>10,000	3585 ± 224	1010 ± 112	>20,000	2.9 ± 0.3
**18**	Ph	H	NO_2_	>10,000	>10,000	>20,000	>20,000	22 ± 2.6
**19**	Ph	OMe	NH_2_	>10,000	>10,000	393 ± 27	>20,000	1 ± 0.30
**20**	CHPh_2_	OMe	H	>10,000	>10,000	7.2 ± 0.41	>20,000	44 ± 3.10
**21**	CHPh_2_	OMe	NO_2_	>10,000	>10,000	260 ± 11	>20,000	0.8 ± 0.04
**22**	-	H	H	>10,000	>10,000	3 ± 2.40	>20,000	5.2 ± 0.31
**23**	-	OMe	H	>10,000	>10,000	174.5 ± 11.40	>20,000	3.29 ± 0.15

**Table 4:** Binding affinity (K_i_) of **TQX-B** derivatives at hA_1_, hA_2A_, and hA_3_ ARs ^a^.

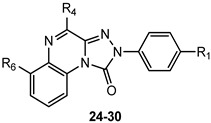						
				K_i_ (nM)		
	R_4_	R_1_	R_6_	hA_1_	hA_2A_	hA_3_
**24**	NHCO-4-pyridyl	H	H	2379 ± 191	188 ± 9.4	6.1 ± 0.5
**25**	NHSO_2_Ph	H	H	>10,000	>10,000	32.2 ± 2.8
**26**	NHSO_2_Ph	OMe	H	2700 ± 142	>10,000	2.2 ± 0.11
**27**	N(SO_2_Me)_2_	H	H	>10,000	>10,000	5.5 ± 0.4
**28**	NHCONHCH_2_Ph	H	H	12.3 ± 1.2	158.3 ± 15	83.5 ± 4.9
**29**	OCH_2_Ph	H	H	>10,000	>10,000	21 ± 1.8
**30**	OCH_2_Ph	OMe	H	>10,000	>10,000	6.4 ± 0.4

^a^ Only compound **27** was tested at the hA_2B_ AR (cAMP assay, IC_50_ > 10,000 nM).

**Table 5:** Binding affinity (K_i_) of 6-(hetero)arylalkylamino-substituted **TQX-B** derivatives at bovine (b) A_1_ and A_2A_ ARs, and at hA_3_ ARs ^a^.

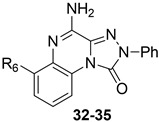				
		K_i_ (nM)		
	R_6_	bA_1_	bA_2A_	hA_3_
**32**	NHCH_2_Ph	730 ± 75.1	6.5 ± 0.7	>1000
**33**	NHCH_2_C_6_H_4_-3-COOH	92 ± 7.8	15.2 ± 1.6	817 ± 79.6
**34**	NHCH_2_-2-furyl	189.4 ± 22.4	8.66 ± 0.9	>1000
**35**	NHCH_2_-3-thienyl	259 ± 16.2	10 ± 1.9	>1000

^a^ Not tested at the hA_2B_ AR.

**Table 6:** Binding affinity (K_i_) of **PTP** derivatives at hA_1_, hA_2A_, and hA_3_ ARs.

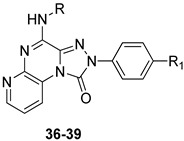							
					K_i_ (nM)		
	R	R_1_	bA_1_	bA_2A_	hA_1_^a^	hA_2A_^a^	hA_3_
**36**	H	H	3.1 ± 0.28	92.6 ± 5.6	n.t.	n.t.	656 ± 41
**37**	H	4-OMe	1102 ± 81	413 ± 34	n.t.	n.t.	158 ± 9.8
**38**	COPh	H	152 ± 10	7100 ± 550	>10,000	2240 ± 230	70.3 ± 6
**39**	COPh	4-OMe	7.15 ± 0.5	>10,000	>10,000	>10,000	4.54 ± 0.2

^a^ n.t. = not tested.

**Table 7:** Binding affinity (K_i_) of **QZ** derivatives at hA_1,_ hA_2A_, hA_3_ ARs and potency (IC_50_) at hA_2B_ and hA_3_ ARs.

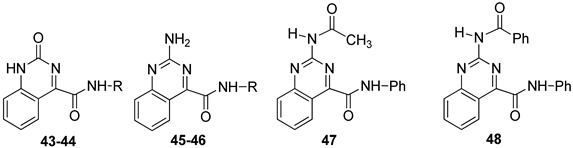						
		K_i_ (nM)			IC_50_ (nM) cAMP	
	R	hA_1_	hA_2A_	hA_3_	hA_2B_	hA_3_^a^
**43**	Ph	>1000	>1000	50 ± 4	>1000	238 ± 21
**44**	C_6_H_4_-4-OMe	>1000	>1000	19.5 ± 2.2	>1000	125 ± 10
**45**	Ph	>1000	>1000	350 ± 40	>1000	n.t.
**46**	C_6_H_4_-4-OMe	>1000	>1000	87.5 ± 6.6	>1000	n.t.
**47**		>1000	>1000	25.3 ± 2.8	>1000	140 ± 13
**48**		>1000	>1000	182 ± 10	>1000	n.t.

^a^ n.t. = not tested.

**Table 8:** Binding affinity (K_i_) of **PHTZ** derivatives at hA_1,_ hA_2A_, hA_3_ ARs and potency (IC_50_) at hA_2B_ and hA_3_ ARs.

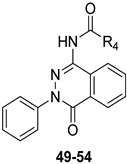						
		K_i_ (nM)			IC_50_ (nM) cAMP	
	R_4_	hA_1_	hA_2A_	hA_3_	hA_2B_^a^	hA_3_^a^
**49**	C_6_H_5_	>10,000	>10,000	1100 ± 100	n.t.	n.t.
**50**	NHPh	>10,000	>10,000	178.4 ± 17	n.t.	n.t.
**51**	NHC_6_H_4-_2-OMe	>10,000	>10,000	8.9 ± 1	>10,000	17 ± 1.6
**52**	NHC_6_H_4-_3-OMe	>10,000	>10,000	9.75 ± 0.25	>10,000	18 ± 2
**53**	NHC_6_H_3_-2,5-OMe	>10,000	>10,000	0.776 ± 0.037	>10,000	8.25 ± 0.6
**54**	NHCH_2_Ph	>10,000	>10,000	29.6 ± 3	>10,000	1.15 ± 002

^a^ n.t. = not tested.

**Table 9:** Binding affinity (K_i_) of **TPZ** derivatives at hA_1,_ hA_2A_, hA_3_ ARs.

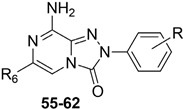					
			K_i_ (nM)		
	R_6_	R	hA_1_	hA_2A_	hA_3_
**55**	Me	H	67 ± 8	485 ± 39	4370 ± 355
**56**	Ph	H	13 ± 1	10 ± 3	11 ± 2
**57**	Ph	4-OMe	20 ± 5	78 ± 18	117 ± 26
**58**	Ph	4-NO_2_	8.1 ± 2.5	402 ± 91	>30,000
**59**	C_6_H_4_-4-OMe	H	>30,000	7.2 ± 1.8	>30,000
**60**	C_6_H_4_-4-OEt	H	>30,000	2.9 ± 0.5	>30,000
**61**	C_6_H_4_-4-O-propargyl	H	>30,000	10.6 ± 1.3	>30,000
**62**	C_6_H_4_-4-O-i-propyl	H	>30,000	7.4 ± 0.9	>30,000

**Table 10:** Binding affinity (K_i_) of **TPZ** derivatives at hA_1,_ hA_2A_, and hA_3_ ARs.

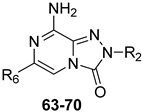					
			K_i_ (nM)		
	R_6_	R_2_	hA_1_	hA_2A_	hA_3_
**63**	2-furyl	Ph	13 ± 2	8.4 ± 0.9	120 ± 18
**64**	C_6_H_4_-4-NO_2_	Ph	7834 ± 597	7.2 ± 1.6	16,421 ± 3505
**65**	C_6_H_4_-4-Br	Ph	>30,000	10.6 ± 2.5	705.4 ± 139.5
**66**	C_6_H_4_-3-Cl	Ph	4.7 ± 1.1	6.3 ± 1	>30,000
**67**	C_6_H_4_-4-Cl	Ph	14.3 ± 3.6	10.9 ± 2.7	>30,000
**68**	Ph	CH_2_Ph	2.4 ± 0.5	4.4 ± 0.1	223.7 ± 4.8
**69**	2-furyl	CH_2_Ph	13.7 ± 0.3	2 ± 0.1	1131 ± 132
**70**	2-(5-methylfuryl)	CH_2_Ph	3.7 ± 0.2	4.6 ± 1.3	112 ± 2

**Table 11:** Binding affinity (K_i_) at hA_1_, hA_2A_, and hA_3_ ARs of **TPZ** derivatives featuring basic substituents in the lateral chain.

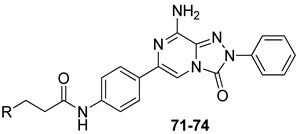				
		K_i_ (nM)		
	R	hA_1_	hA_2A_	hA_3_
**71**	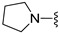	296 ± 3 6	4.31 ± 0.5	1016 ± 165
**72**	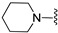	614 ± 145	5.1 ± 1.3	1169 ± 85
**73**	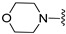	586 ± 164	3.6 ± 1.1	1023 ± 76.7
**74**	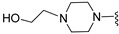	555.5 ± 37	7.27 ± 1.7	2454 ± 335

**Table 12:** Binding affinity (K_i_) of the antioxidant-based **TPZ** derivatives at hA_1_, hA_2A_, and hA_3_ ARs.

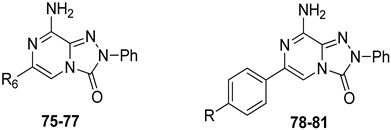				
		K_i_ (nM)		
	R_6_/R	hA_1_	hA_2A_	hA_3_
**75**	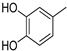	42.6 ± 9.6	5.2 ± 0.5	950 ± 200
**76**	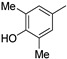	21.3 ± 7	2.5 ± 0.8	100 ± 0.7
**77**	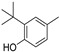	>30,000	8.5 ± 1.4	>30,000
**78**	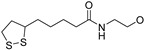	378.6 ± 91	2.4 ± 0.3	4097 ± 812
**79**	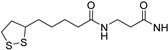	1359 ± 284	36.4 ± 8.2	>30,000
**80**	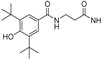	>30,000	54.5 ± 7.1	>30,000
**81**	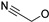	>30,000	8.2 ± 2.3	>30,000

**Table 13:** Biological activity of multitarget **TPZ** derivatives at hARs and inhibition data against hCA I, II, IX, and XII isoforms as determined by a stopped-flow CO_2_ hydrase assay.

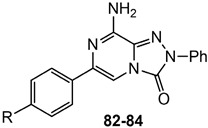								
		K_i_ (nM)			K_i_ (µM)			
	R	hA_1_	hA_2A_	hA_3_	hCA I	hCA II	hCA IX	hCA XII
**82**	SO_2_NH_2_	205 ± 29	856.6 ± 188	14,830 ± 320	8.023	0.703	8.920	0.602
**83**	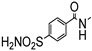	4189 ± 59.5	6.4 ± 1.5	>30,000	8.351	0.046	0.466	0.006
**84**	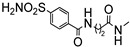	1074 ± 254	108 ± 25	>30,000	0.052	0.0086	0.005	0.027

**Table 14:** Binding affinity (K_i_) of **PQ-A** derivatives at hA_1_, hA_2A_, and hA_3_ ARs ^a^.

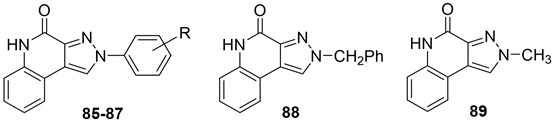				
		K_i_ (nM)		
	R	hA_1_	hA_2A_	hA_3_
**85**	H	203 ± 12	>10,000	30.8 ± 2.6
**86**	4-Me	29 ± 0.5	>10,000	3.2 ± 0.2
**87**	4-OMe	176.4 ± 8.8	>10,000	3.2 ± 0.2
**88**	-	>10,000	>10,000	74.5 ± 5.3
**89**	-	>10,000	>10,000	>1000

^a^ Not tested at hA_2B_ AR.

**Table 15:** Binding affinities (K_i_) of **PQ-B** derivatives at hA_1_, hA_2A_, and hA_3_ ARs ^a^.

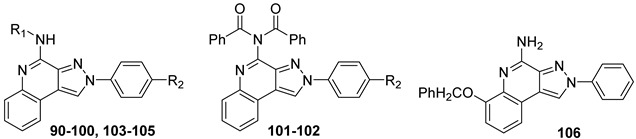					
			K_i_ (nM)		
	R_1_	R_2_	hA_1_	hA_2A_	hA_3_
**90**	H	4-OMe	40 ± 3.1	1060 ± 96	8.9 ± 0.6
**91**	COMe	H	>10,000	>10,000	48.2 ± 3.5
**92**	COMe	3-Me	203 ± 15	>10,000	31 ± 2.4
**93**	COPh	H	>10,000	>10,000	2.1 ± 0.1
**94**	COPh	4-OMe	250 ± 13	>10,000	3.4 ± 0.2
**95**	COCH_2_Ph	H	>10,000	>10,000	9.9 ± 0.8
**96**	COCH_2_Ph	4-OMe	201 ± 12	>10,000	4.5 ± 0.6
**97**	COCHPh_2_	H	>10,000	>10,000	8.9 ± 0.6
**98**	COCHPh_2_	4-OMe	>10,000	>10,000	9.0 ± 0.5
**99**	CONHCH_2_Ph	H	>10,000	>10,000	8.3 ± 0.7
**100**	CONHCH_2_Ph	3-Me	6800 ± 510	>10,000	3.35 ± 0.2
**101**	-	H	>10,000	>10,000	6.1 ± 0.5
**102**	-	4-OMe	>10,000	>10,000	17.2 ± 1.4
**103**	CO(2-furyl)	H	>10,000	>10,000	3.4 ± 0.3
**104**	CO(4-pyridyl)	H	>10,000	>10,000	5.0 ± 0.6
**105**	CO(3-Me-C_6_H_4_)	H	>10,000	>10,000	6.3 ± 0.7
**106**	-	-	140 ± 12	>10,000	>1000

^a^ Not tested at hA_2B_ AR.

**Table 16:** Binding affinity (K_i_ or I%) of **PP-7-oxo** derivatives at h A_1_, hA_2A_, and hA_3_ ARs and potency (cAMP assays I%) at hA_2B_ AR.

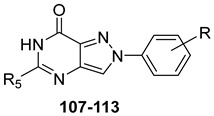						
			K_i_ (nM) or I% (@ 1 µM)			I% (@ 1 µM)
	R_5_	R	hA_1_	hA_2A_	hA_3_	hA_2B_
**107**	H	H	1%	1%	185 ± 19	3%
**108**	Me	H	9%	1%	16 ± 2	2%
**109**	Ph	H	10%	22%	10%	4%
**110**	CH_2_Ph	H	11%	1%	900 ± 95	4%
**111**	Me	4-OMe	5%	1%	1.2 ± 0.1	2%
**112**	Me	3-Me	4%	1%	72 ± 8	2%
**113**	Me	4-Me	1%	1%	10 ± 1	4%

**Table 17:** Binding affinity (K_i_ or I%) of **PP-7-amino** derivatives at hA_1_, hA_2A_, and hA_3_ ARs and potency (cAMP assays, IC_50_ or I%) at hA_2B_ AR.

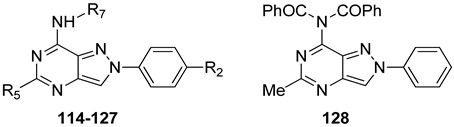							
				K_i_ (nM) or I% (@ 1 µM)			IC_50_ (nM) or I% (@ 1 µM)
	R_5_	R_2_	R_7_	hA_1_	hA_2A_	hA_3_	hA_2B_
**114**	Me	H	H	70 ± 6	246 ± 23	40%	320 ± 35
**115**	Me	4-OMe	H	30%	1%	38%	2%
**116**	Ph	H	H	75 ± 7	325 ± 34	48%	440 ± 43
**117**	2-thienyl	H	H	52 ± 3	115 ± 14	9.7 ± 0.9	27%
**118**	C_6_H_4_-4-OMe	4-OMe	H	1%	3%	17 ± 2	1%
**119**	Me	H	COPh	30%	1%	5.6 ± 0.5	2%
**120**	Me	H	CO(C_6_H_4_-4-OMe)	4%	1%	2.4 ± 0.2	1%
**121**	Ph	H	COCH_2_Ph	5%	5%	18 ± 2	2%
**122**	Ph	4-OMe	COPh	3%	1%	18 ± 2	2%
**123**	Ph	4-OMe	COCH_2_Ph	29%	18%	24 ± 3	2%
**124**	2-thienyl	H	COPh	1%	1%	2.12 ± 0.15	1%
**125**	2-thienyl	H	CO(C_6_H_4_-4-OMe)	1%	1%	0.027 ± 0.003	1%
**126**	2-thienyl	H	CO-3-pyridyl	764 ± 68	3%	0.41 ± 0.04	1%
**127**	C_6_H_4_-4-OMe	H	CO(C_6_H_4_-4-OMe)	3%	1%	1.31 ± 0.12	1%
**128**				6%	1%	33 ± 4	5%

**Table 18:** Binding affinity (K_i_ or I%) of **PP-7-amino** derivatives at h A_1_, hA_2A_, and hA_3_ ARs and potency (cAMP assays, IC_50_ or I%) at hA_2B_ AR.

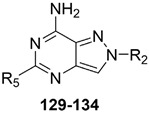						
			K_i_ (nM) or I% (@ 1 µM)			IC_50_ (nM) or I% (@ 1 µM)
	R_5_	R_2_	hA_1_	hA_2A_	hA_3_	hA_2B_
**129**	(CH_2_)_3_Ph	Ph	5.31 ± 0.42	55 ± 5	12%	42%
**130**	(CH_2_)_3_(C_6_H_4_-3-OH)	Ph	0.22 ± 0.03	146 ± 15	46%	314 ± 26
**131**	2-furyl	Ph	206 ± 17	195 ± 14	39%	1%
**132**	2-furyl	CH_2_(C_6_H_4_-2-OMe)	98 ± 8	5.37 ± 0.39	196 ± 17	512 ± 49
**133**	2-(5-methylfuryl)	CH_2_Ph	136 ± 12	9.23 ± 0.85	269 ± 25	20%
**134**	2-(5-methylfuryl)	CH_2_(C_6_H_4_-2-OH)	120 ± 11	5.26 ± 0.47	88 ± 6	293 ± 26

**Table 19:** Binding affinity (K_i_ or I%) of **IP** derivatives at hA_1_, hA_2A_, and hA_3_ ARs and potency (cAMP assays, I%) at hA_2B_ AR.

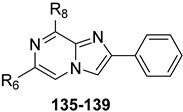						
			K_i_ (nM) or I% (@ 1 µM)			I% (@ 1 µM)
	R_6_	R_8_	hA_1_	hA_2A_	hA_3_	hA_2B_
**135**	H	NHCOPh	18%	1%	52 ± 5	2%
**136**	Ph	NHCOPh	1%	1%	82 ± 7	1%
**137**	Ph	NHCO(C_6_H_4_-4-OMe)	6%	8%	25 ± 3	1%
**138**	Ph	NHCO(C_6_H_4_-4-F)	1%	1%	38 ± 4	1%
**139**	Ph	NHCO(3-pyridyl)	11%	1%	54 ± 6	3%

**Table 20:** Binding affinity (K_i_ or I%) of **TP-7-oxo** derivatives at h A_1_, hA_2A_, and hA_3_ ARs and potency (cAMP assays, I%) at hA_2B_ AR.

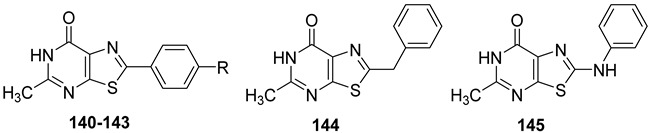					
		K_i_ (nM) or I% (@ 1 µM)			I% (@ 1 µM)
	R	hA_1_	hA_2A_	hA_3_	hA_2B_
**140**	Cl	1%	1%	18 ± 2	1%
**141**	OMe	10%	1%	38 ± 4	3%
**142**	Me	17%	2%	33 ± 4	3%
**143**	OH	1%	1%	15%	1%
**144**	-	11%	1%	427 ± 46	1%
**145**	-	24%	14%	45%	1%

**Table 21:** Binding affinity (K_i_) of 2-furyl-**TP-7-amino** derivatives at hA_1_, hA_2A_, and hA_3_ ARs and potency (cAMP assays, IC_50_) at hA_2B_ AR.

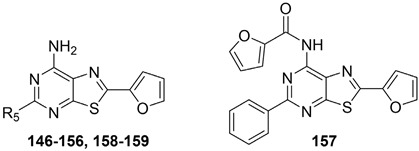					
		K_i_ (nM) or KH * (fM) or KL ** (nM)			IC_50_ (nM)
	R_5_	hA_1_	hA_2A_	hA_3_	hA_2B_
**146**	NHCH_2_CH_2_(C_6_H_4_-4-OH)	37 ± 4	18 ± 2	1884 ± 167	482 ± 41
**147**	NHCH_2_(C_6_H_4_-2-OMe)	3.54 ± 0.32	3.55 ± 0.42 * 6.45 ± 0.57 **	36 ± 3	313 ± 29
**148**	NHCH_2_(C_6_H_4_-3-OMe)	8.16 ± 0.72	5.31 ± 0.52 * 26 ± 2 **	92 ± 8	452 ± 42
**149**	NHCH_2_(2-thienyl)	12.5 ± 1.1	10.7 ± 1.0 * 3.82 ± 0.31 **	6.43 ± 0.58	75 ± 8
**150**	NHCH_2_(2-furyl)	38 ± 4	39 ± 4 * 1.73 ± 0.15 **	4.72 ± 0.38	82 ± 9
**151**	NHCH_2_(3-pyridyl)	7.12 ± 0.65	217 ± 19 * 0.68 ± 0.05 **	18.2 ± 1.7	109 ± 11
**152**	NHCH_2_CH_2_(2-thienyl)	4.92 ± 0.37	10.6 ± 0.9 * 18 ± 2 **	65 ± 6	112 ± 11
**153**	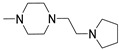	638 ± 56	15.1 ± 1.3	>10,000	>10,000
**154**	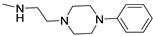	102 ± 9	8.62 ± 0.74	>10,000	>10,000
**155**	Ph	33 ± 2	3 ± 0.04	15 ± 2.9	>10,000
**156**	2-furyl	69 ± 15	3.4 ± 0.9	99 ± 15	>10,000
**157**	-	265 ± 63	428 ± 12	4 ± 0.51	>10,000
**158**	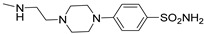	89 ± 8	2.02 ± 0.18	>10,000	>10,000
**159**	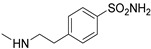	1326 ± 256	5.73 ± 0.48	1874 ± 158	34 ± 3

**Table 22:** Binding affinity (K_i_) of **TP-7-amino** derivatives at hA_1_, hA_2A_, and hA_3_ ARs and potency (cAMP assays, IC_50_) at hA_2B_ AR.

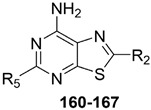						
			K_i_ (nM)			IC_50_ (nM)
	R_2_	R_5_	hA_1_	hA_2A_	hA_3_	hA_2B_
**160**	Ph	NHCH_2_(C_6_H_4_-2-OMe)	102 ± 1.1	4.72 ± 0.46	692 ± 67	2622 ± 224
**161**	pyrazin-2-yl	NHCH_2_(C_6_H_4_-2-OMe)	112 ± 10	79 ± 7	725 ± 67	4017 ± 385
**162**	Me	NHCH_2_(C_6_H_4_-2-OMe)	281 ± 26	135 ± 11	782 ± 68	5538 ± 412
**163**	2-thienyl	NHCH_2_(C_6_H_4_-2-OMe)	17.3 ± 1.5	2.24 ± 0.21	275 ± 22	4571 ± 328
**164**	Ph	2-furyl	67 ± 6.8	1.7 ± 0.2	2.8 ± 0.4	>10,000
**165**	Ph	Ph	148 ± 16	19 ± 6.2	84 ± 13	>10,000
**166**	CH_2_(C_6_H_4_-2-F)	2-furyl	1.9 ± 0.08	0.06 ± 0.02	93.1 ± 2.8	384 ± 55
**167**	CH_2_(C_6_H_4_-2-Cl)	2-(5-methylfuryl)	0.5 ± 0.1	0.07 ± 0.006	8.5 ± 1.6	8847 ± 1445

## Data Availability

No new data were created or analyzed in this study.
